# Supplementary figures and images for: Tmprss2 maintains epithelial barrier integrity and transepithelial sodium transport
Source: Life Sci Alliance. 2024 Jan 3;7(3):e202302304. doi: 10.26508/lsa.202302304 (PMC10765116; doi:10.26508/lsa.202302304)

Figure 2A

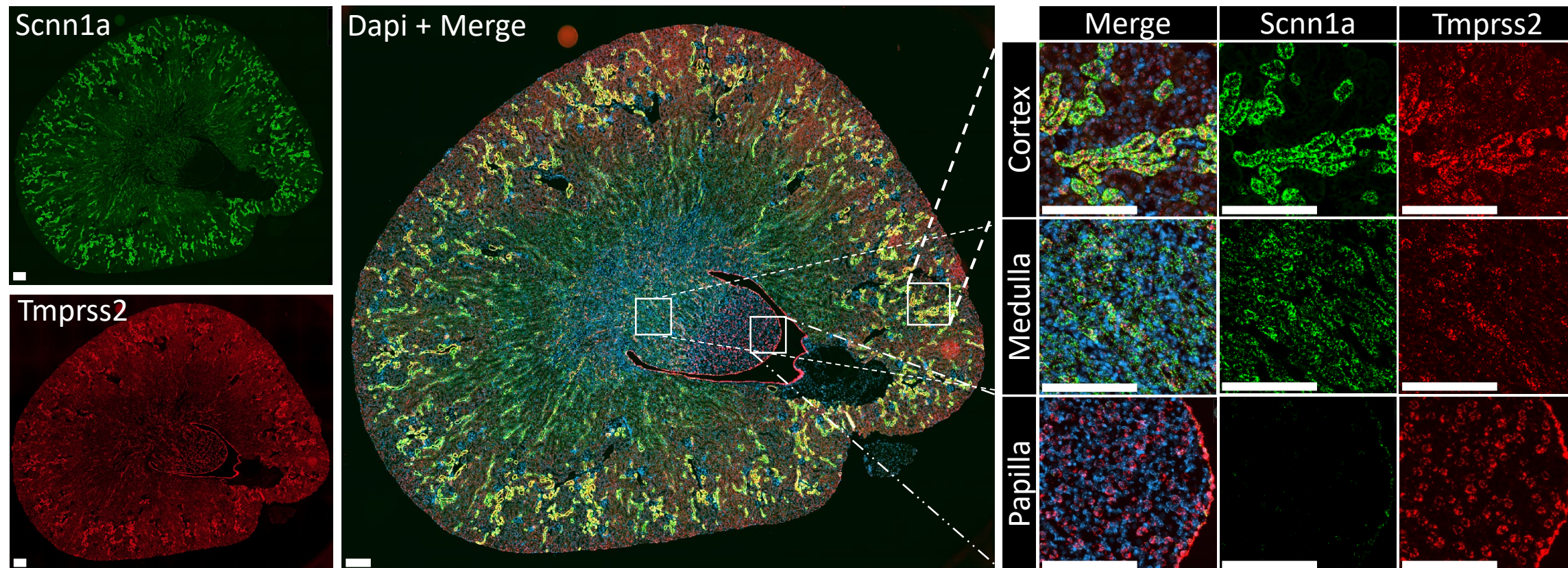

Figure 2B

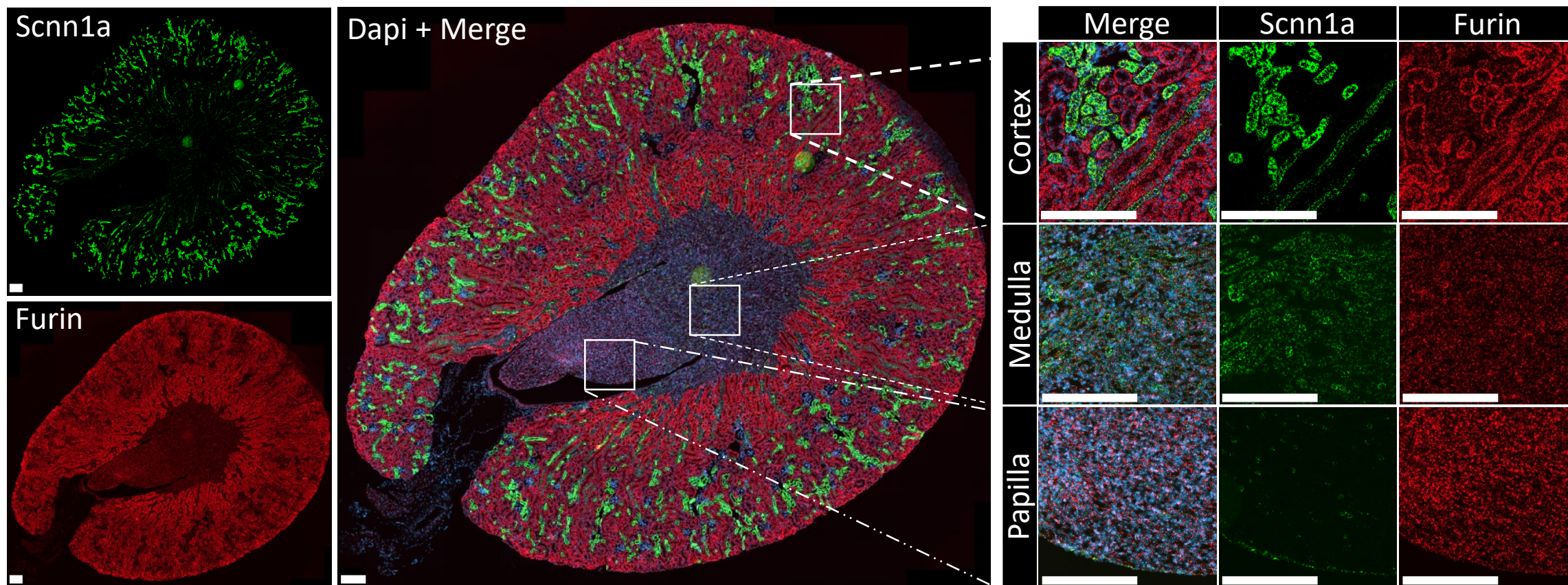

Scale Bar 200um

Supplement: Supplementary file 1 [file LSA-2023-02304_SdataF2.pdf]

Figure S1A

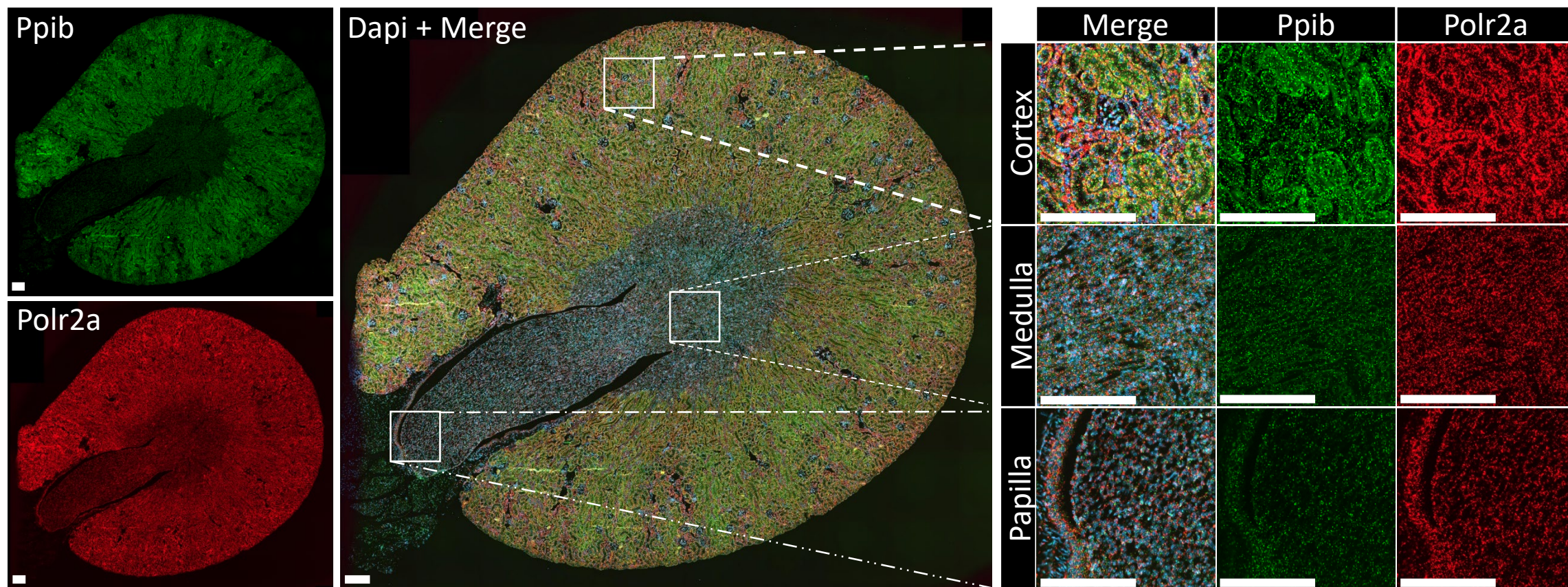

Scale Bar 200um

Figure S1B

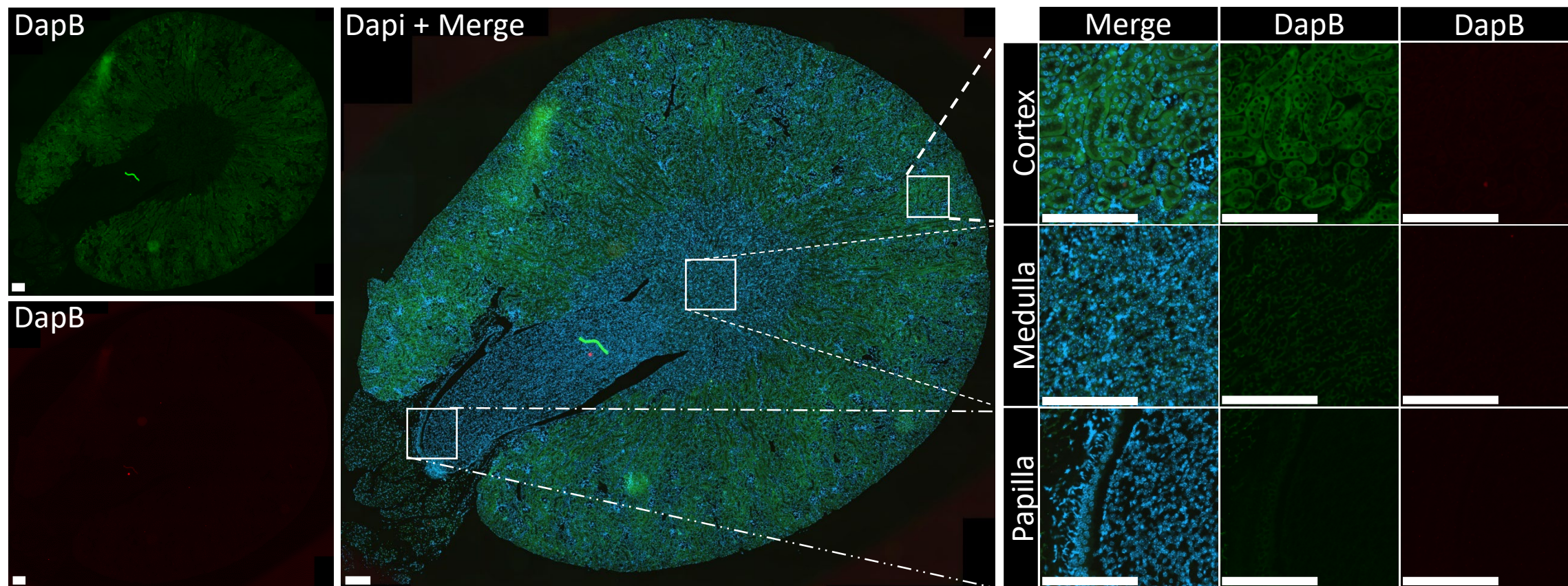

Scale Bar 200um

Supplement: Supplementary file 2 [file LSA-2023-02304_SdataFS1.pdf]

Figure 4C

$\alpha$ ENaC

100 ng/ml 1 ug/ml  
control Dox Dox + -  
1 2 3 4 5 6 7 8 9 10 11 12

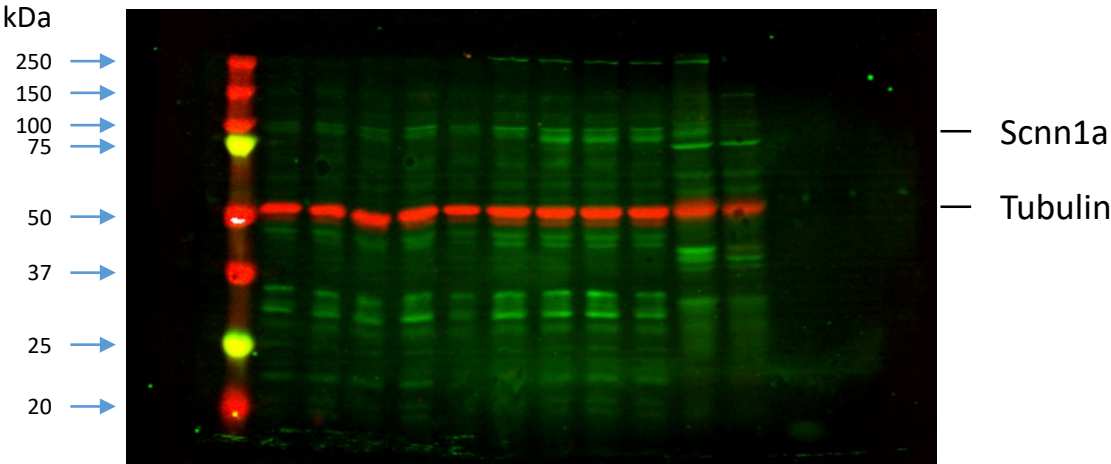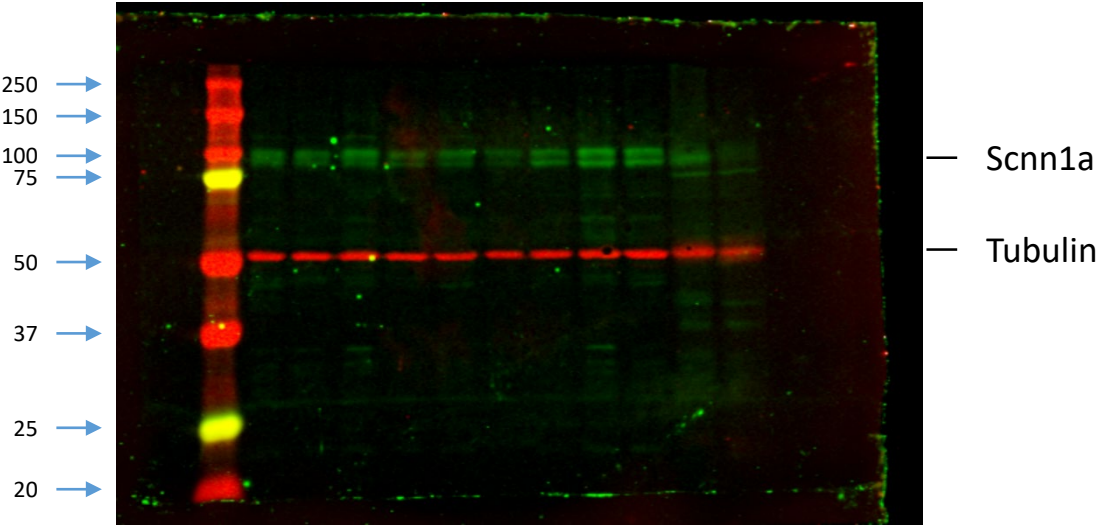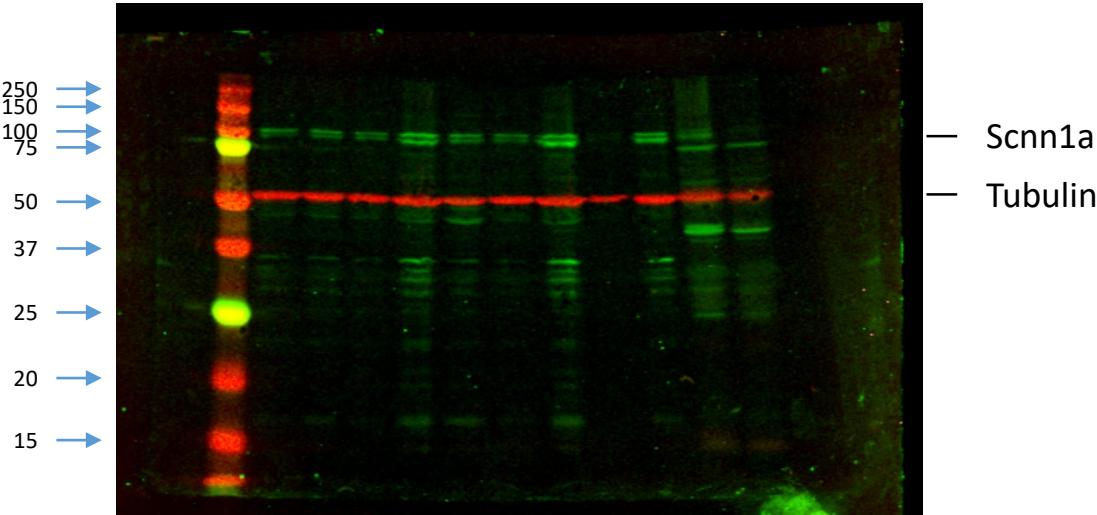

Figure 4C

$\gamma$ ENaC

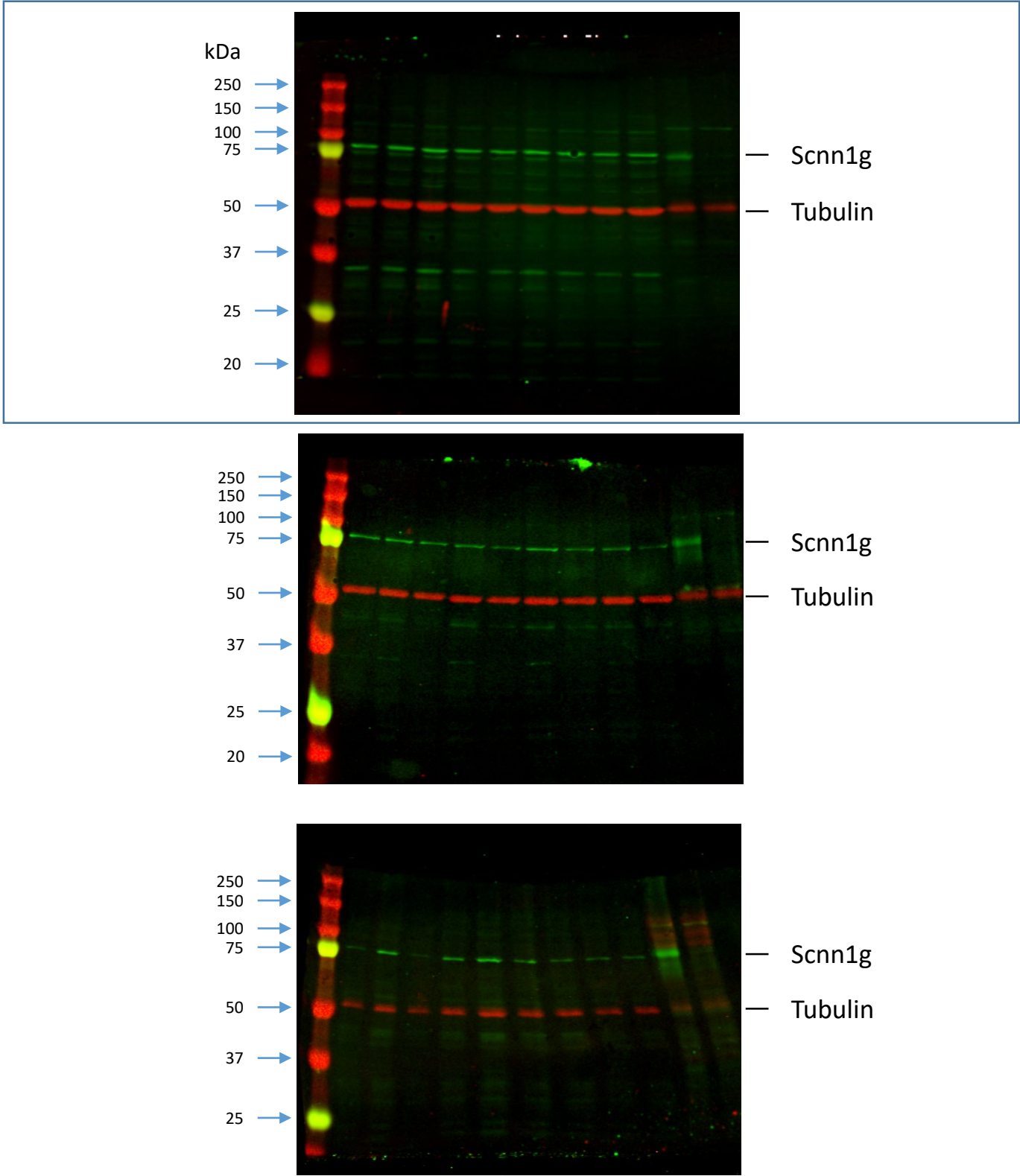

Figure 4C

furin

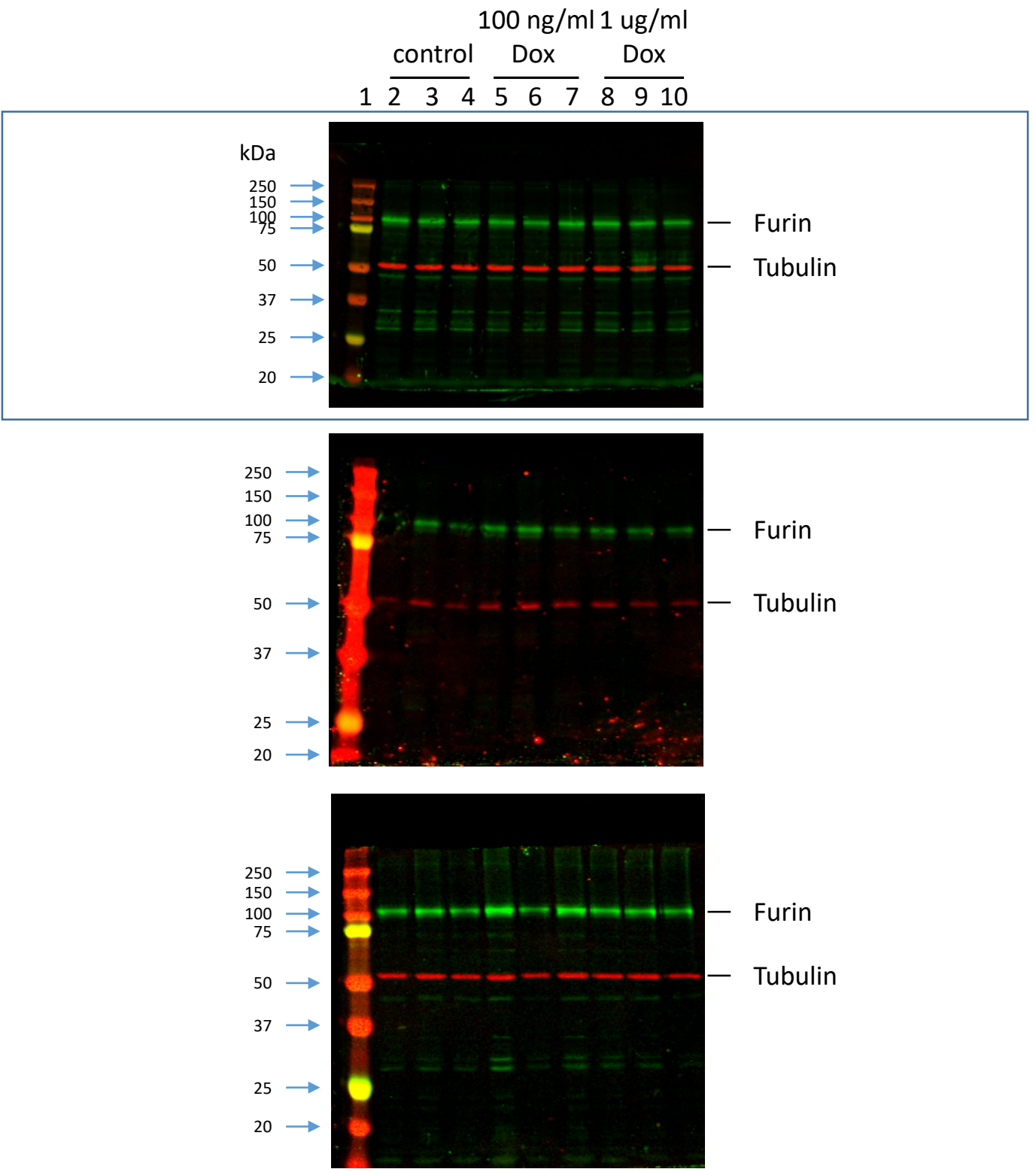

Supplement: Supplementary file 4 [file LSA-2023-02304_SdataF4.pdf]

Figure S3C

$\alpha$ ENaC      aprotinin

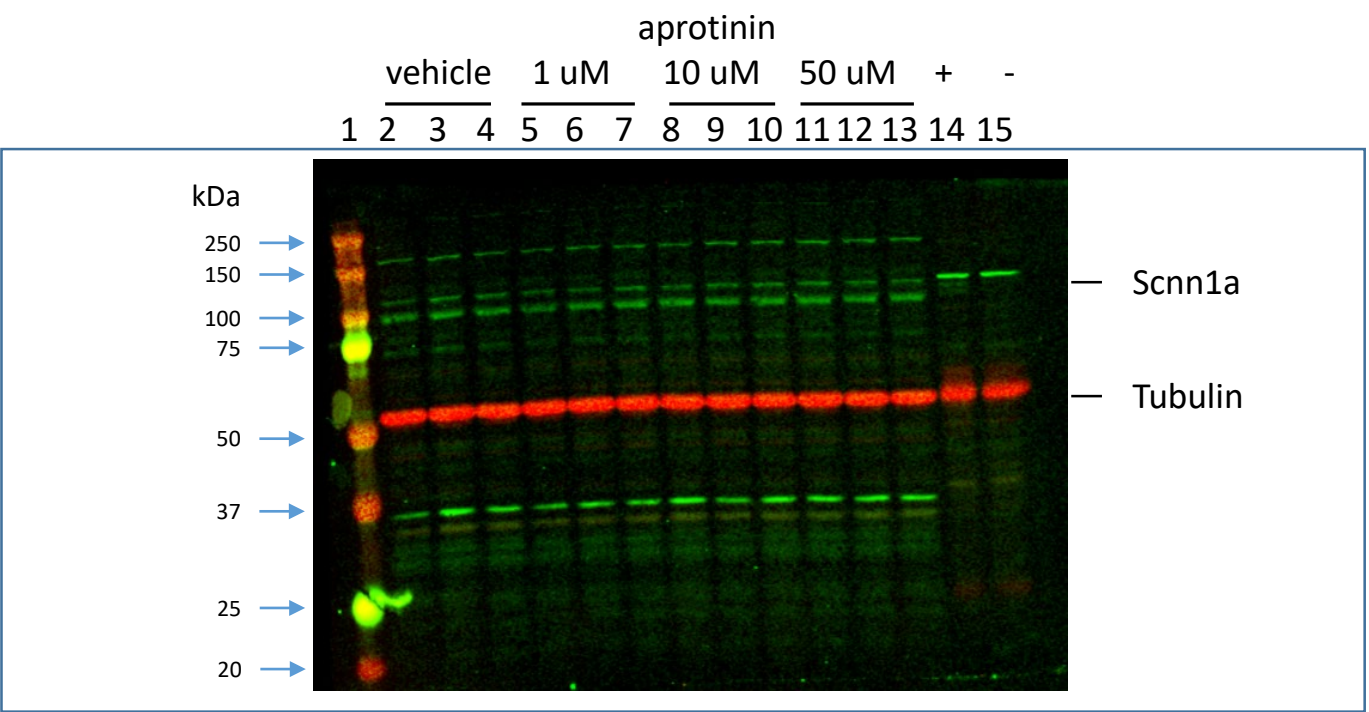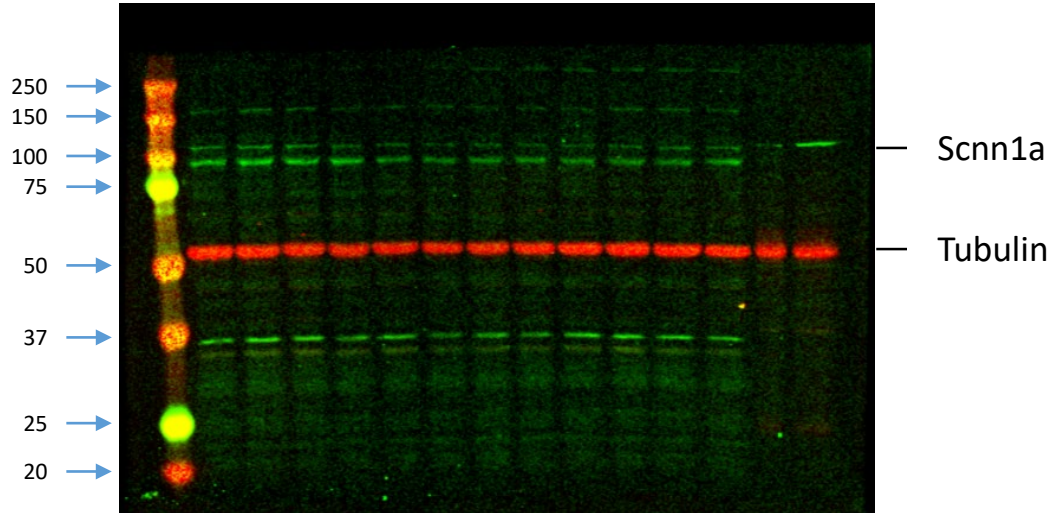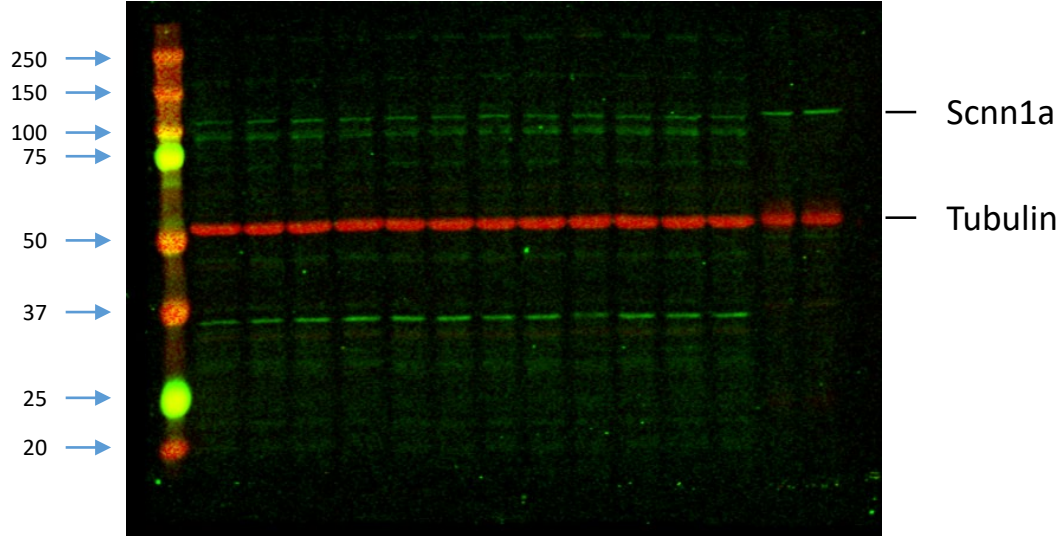

Figure S3C

$\alpha$ ENaC    camostat

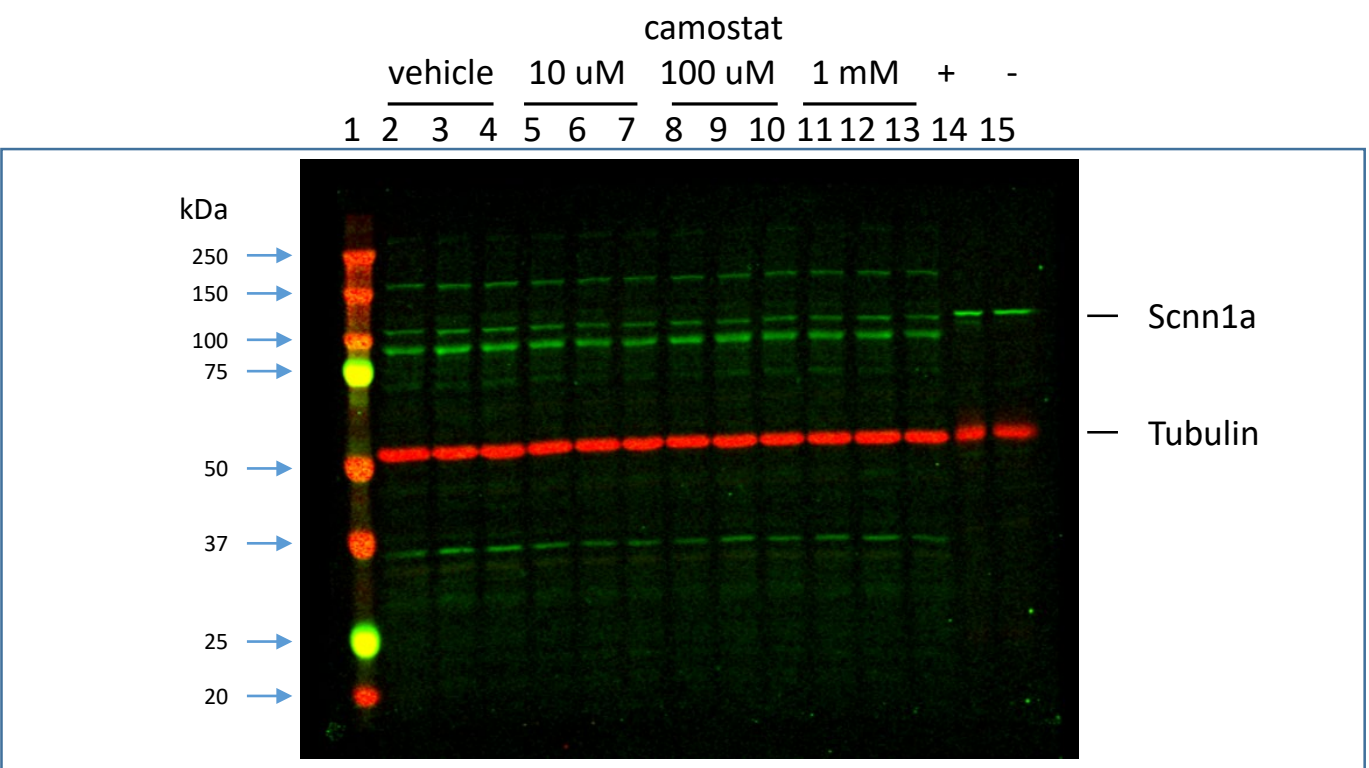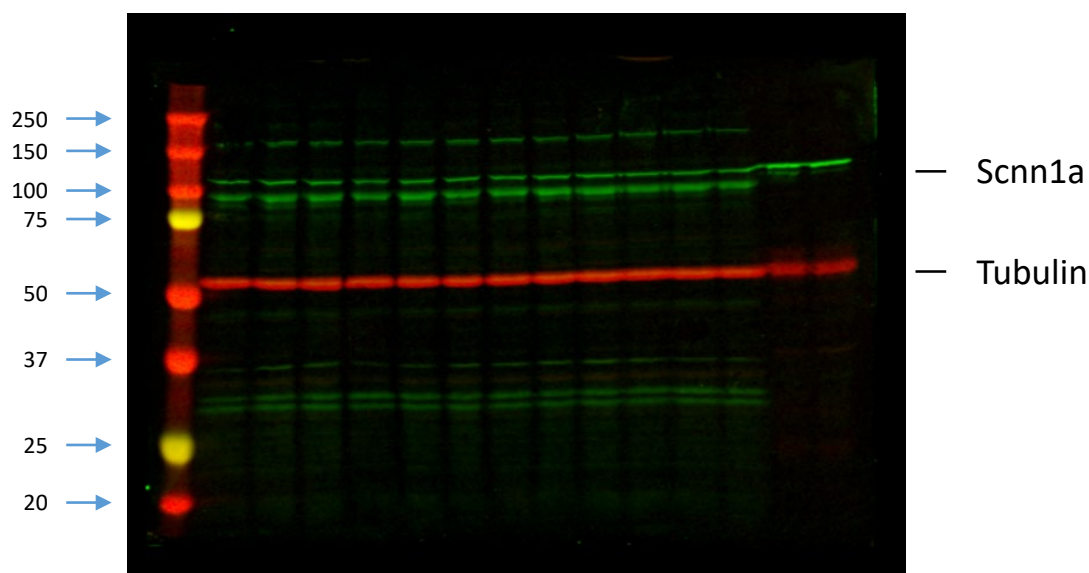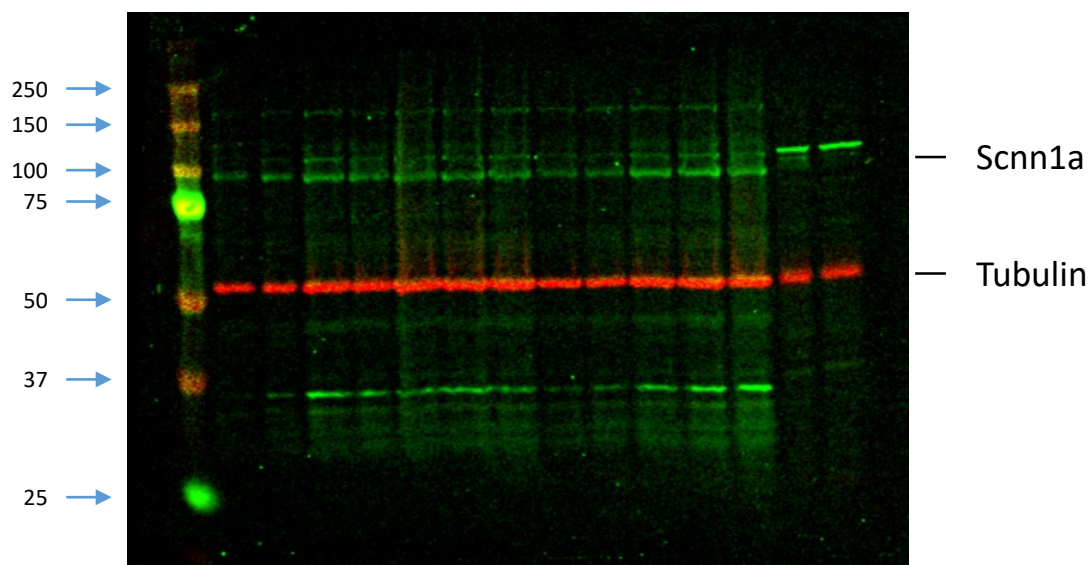

Supplement: Supplementary file 5 [file LSA-2023-02304_SdataFS3.pdf]

Figure 5C

$\alpha$ ENaC

          C1  C2  KO1  KO2  +  -  
          1   2   3   4   5   6   7

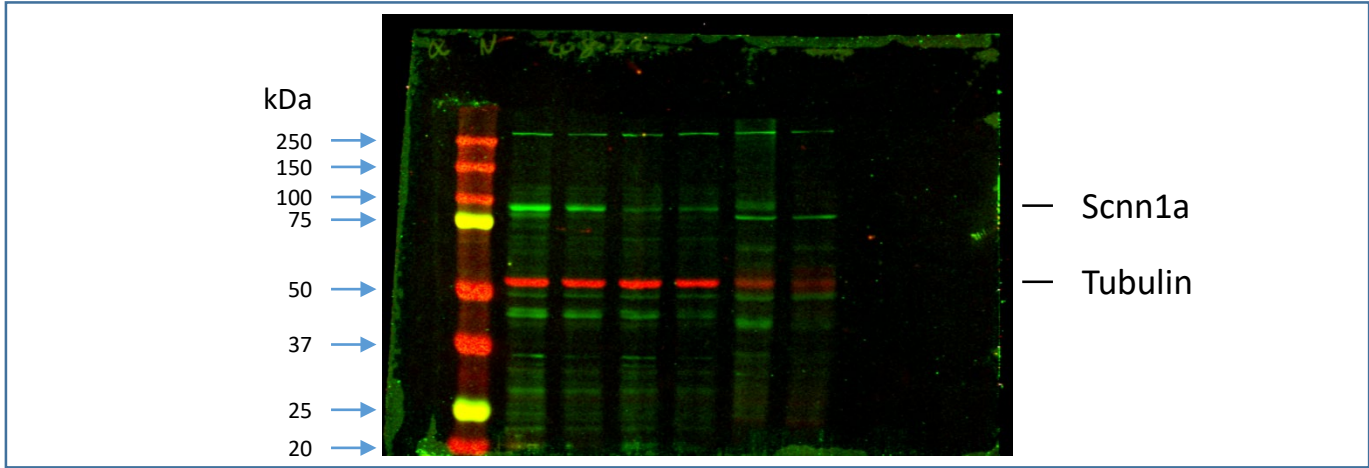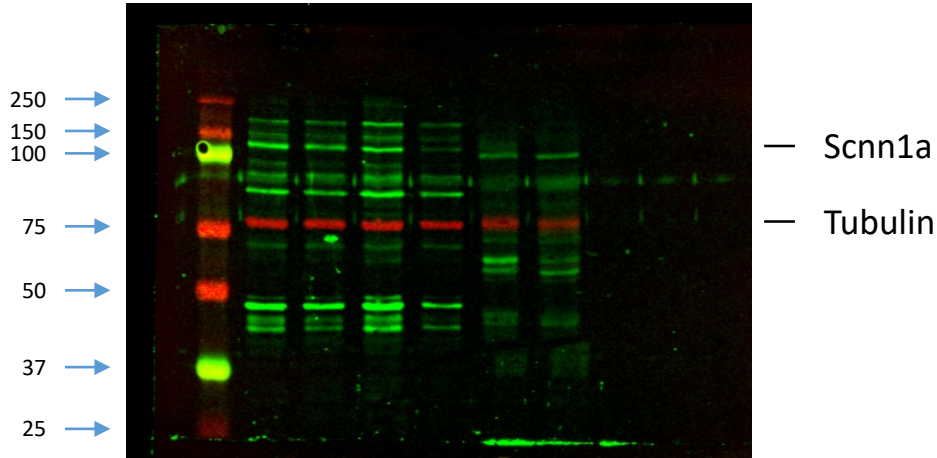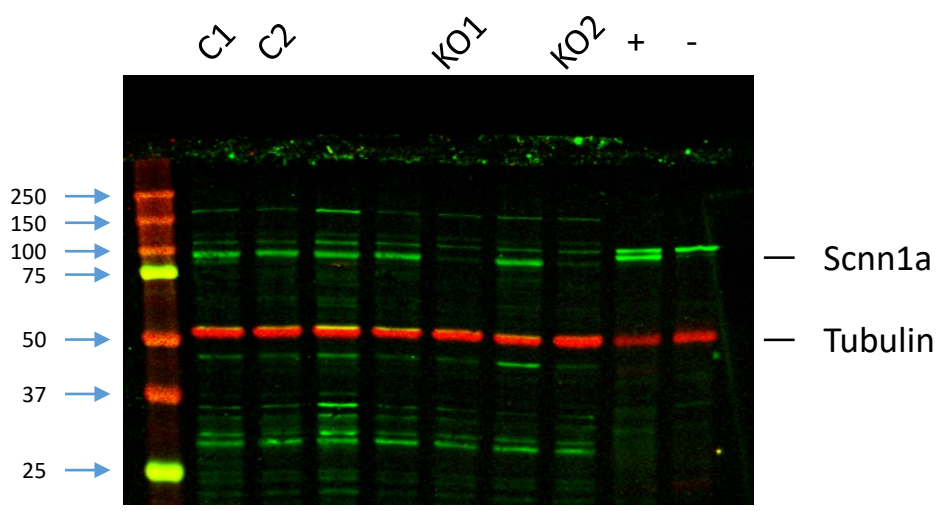

Figure 5C

$\gamma$ ENaC

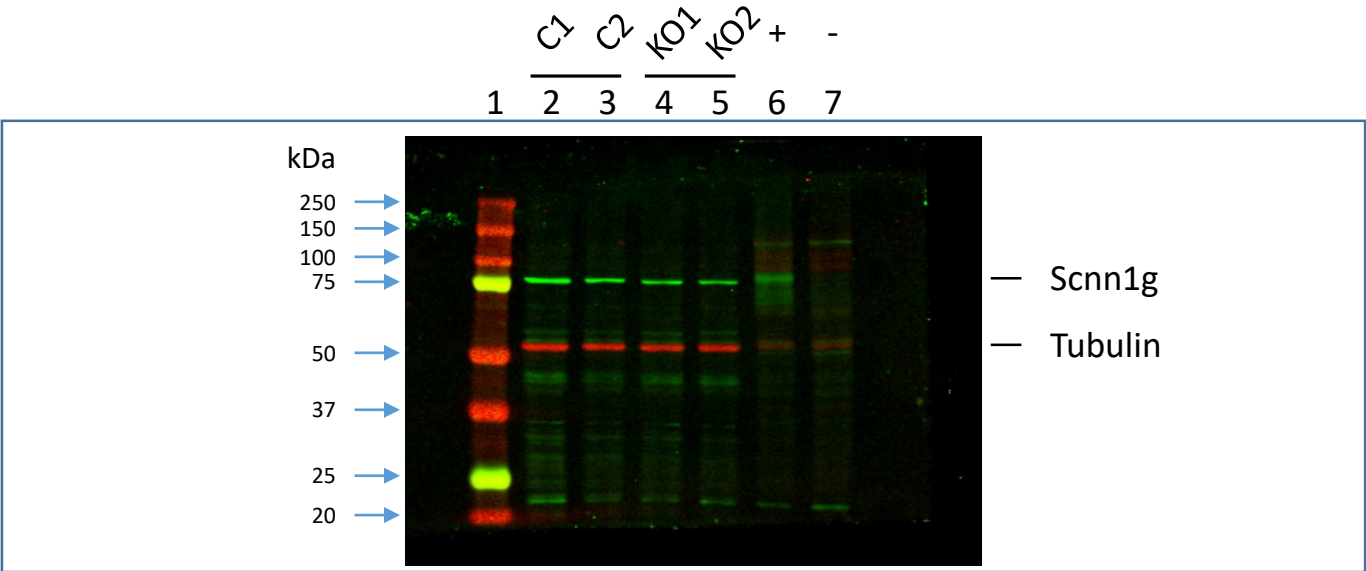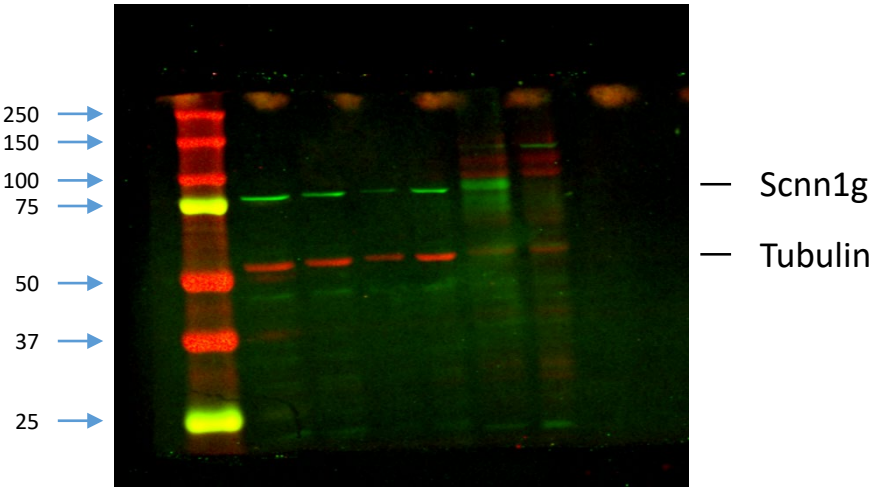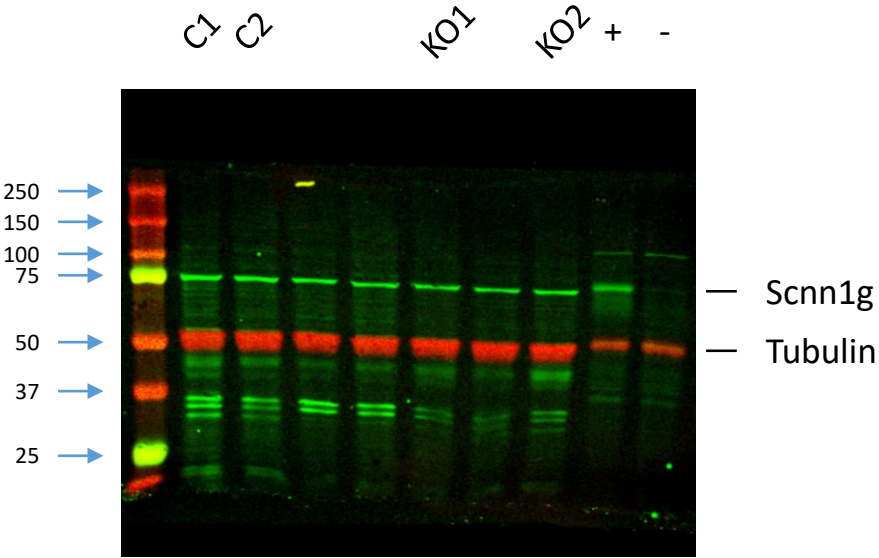

Figure 5C

furin

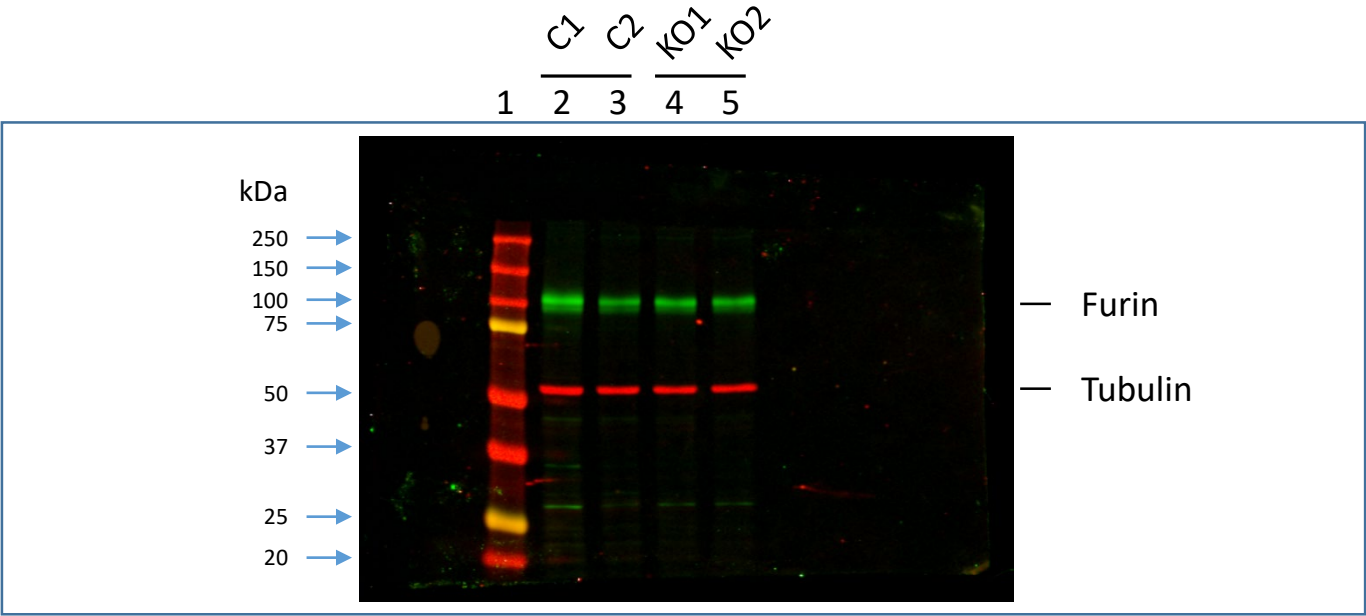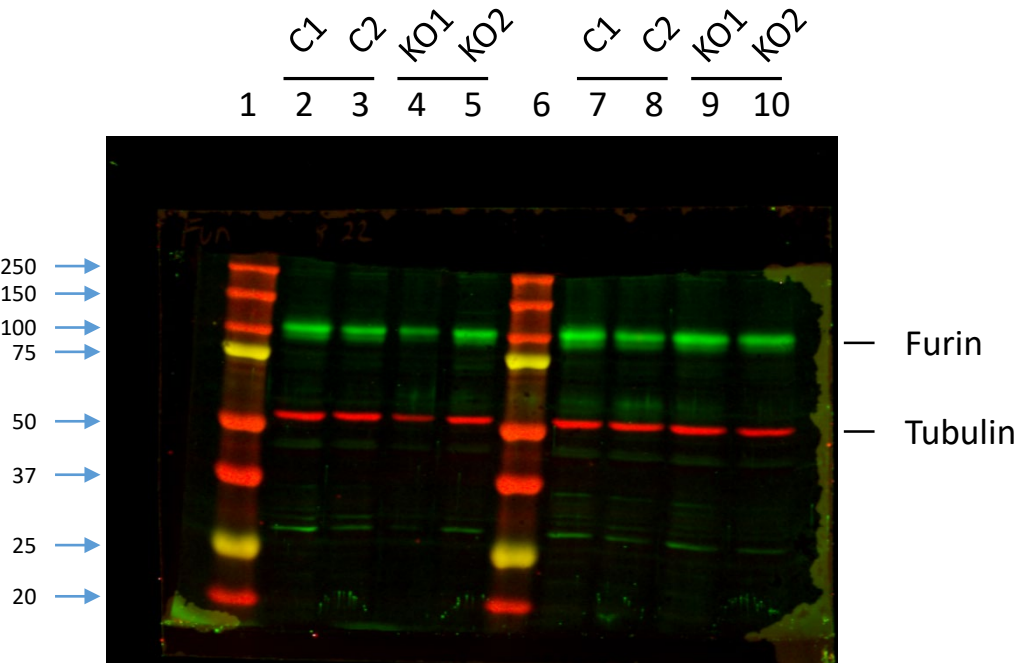

Supplement: Supplementary file 6 [file LSA-2023-02304_SdataF5.pdf]

Figure S5B

Prss8

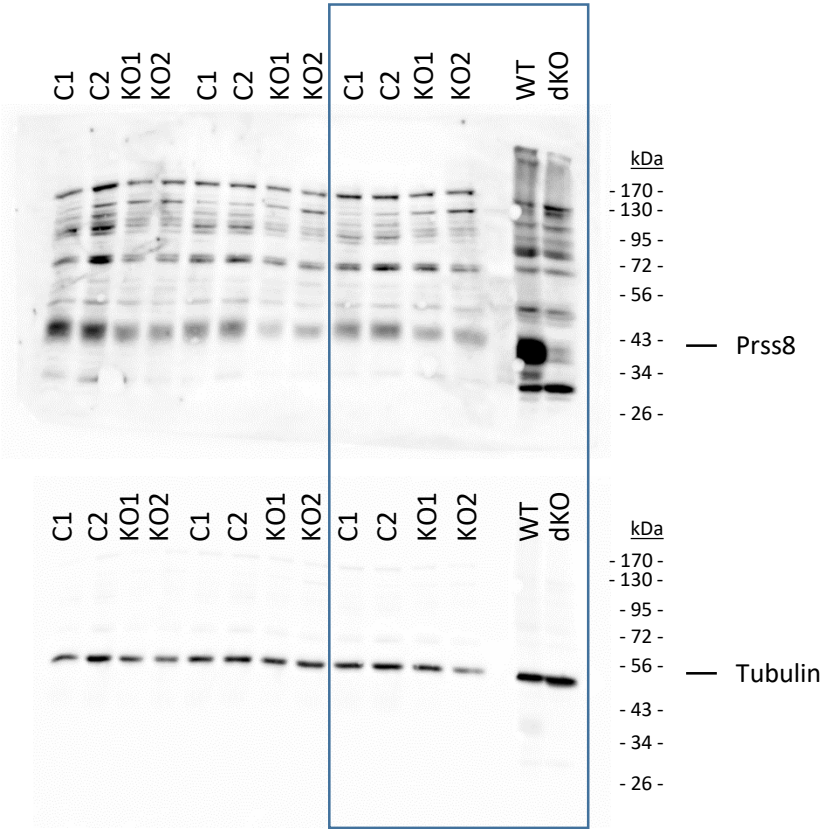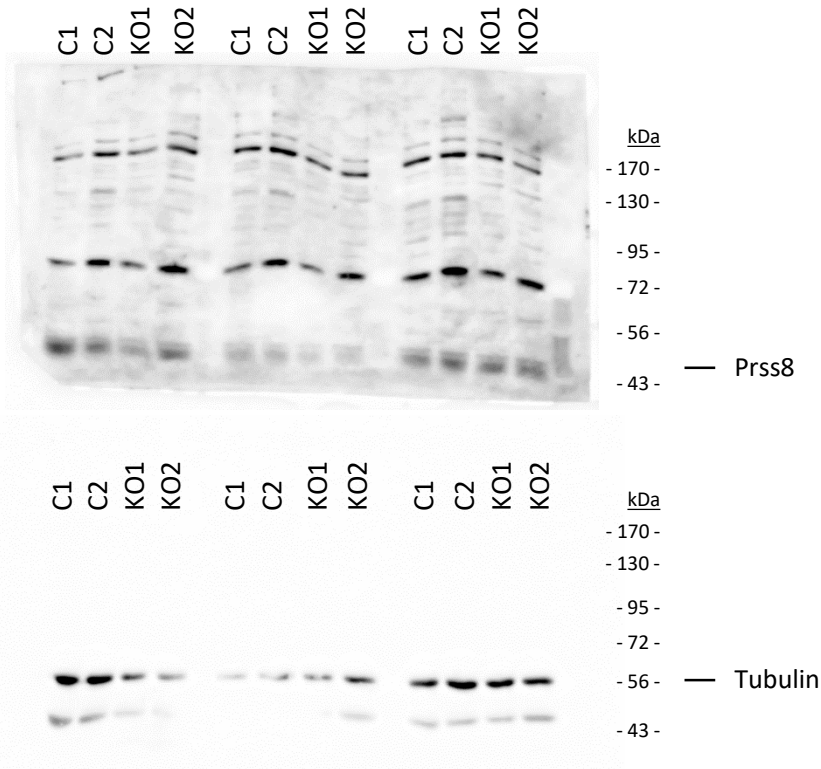

Figure S5D

ZO-1

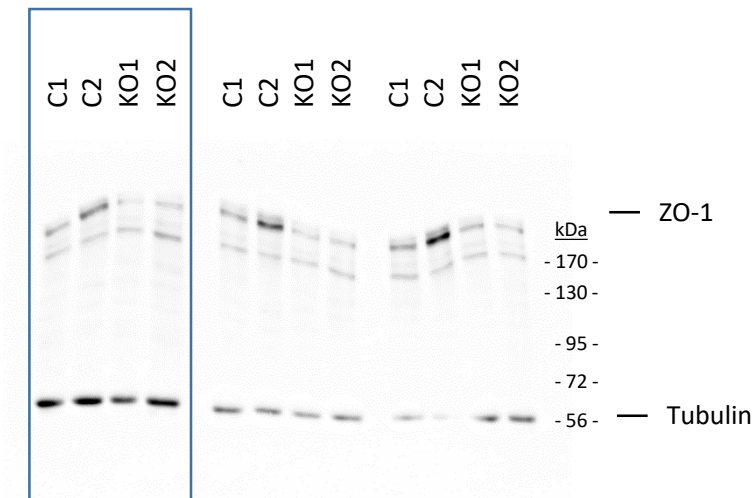

Figure S5F

occludin

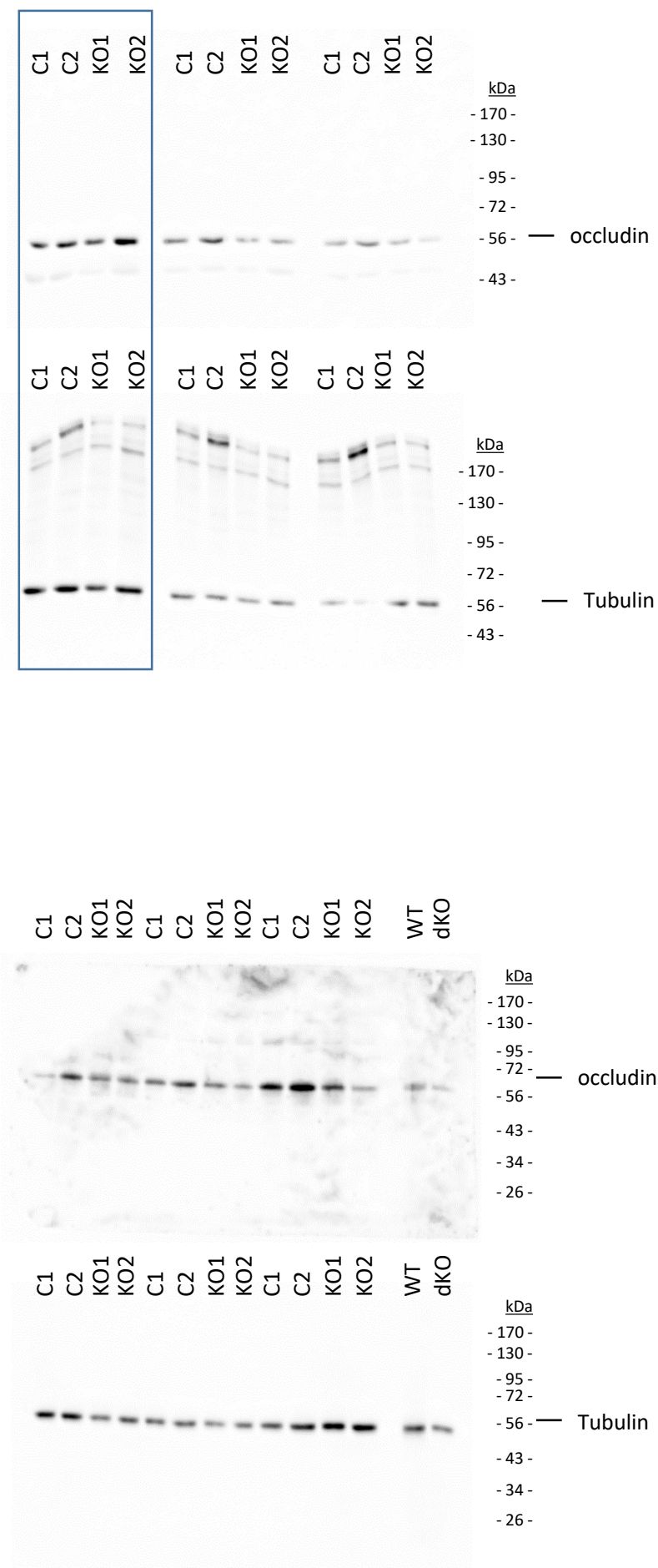

Figure S5H

E-cadherin

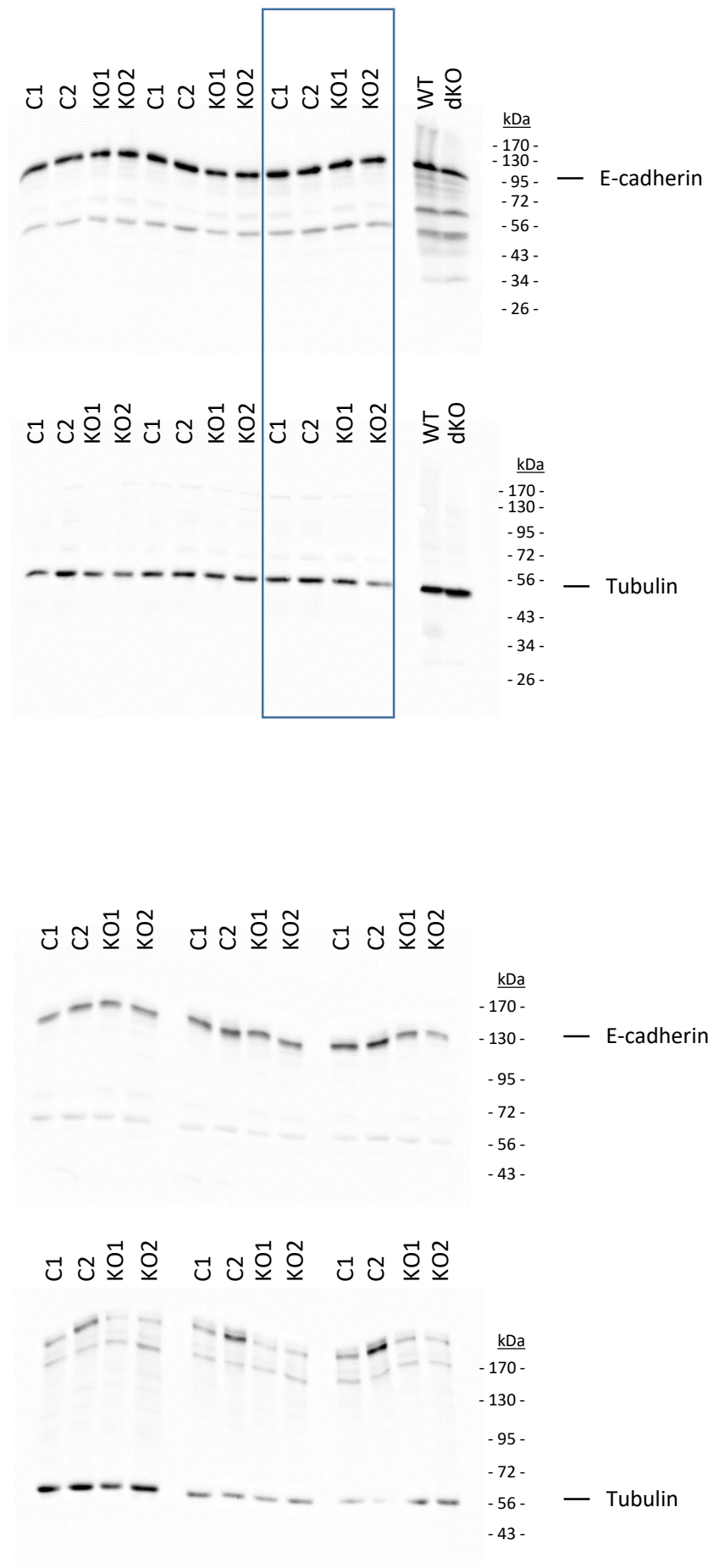

Supplement: Supplementary file 7 [file LSA-2023-02304_SdataFS5.pdf]

Figure 8B

Claudin-2

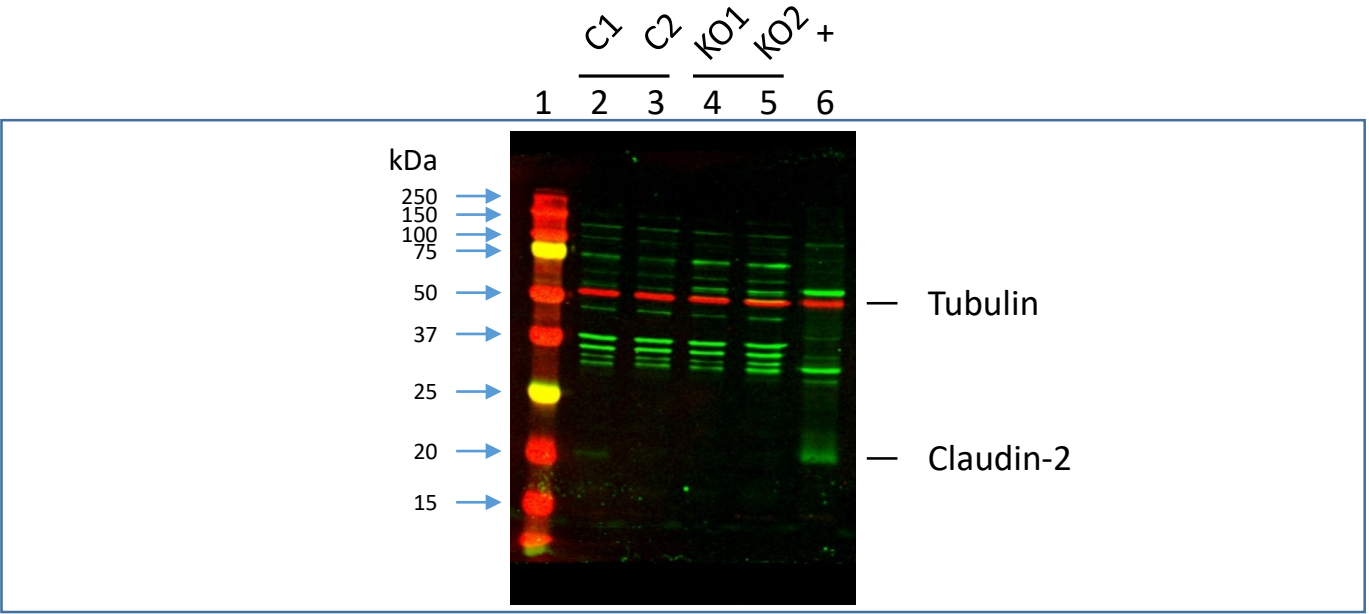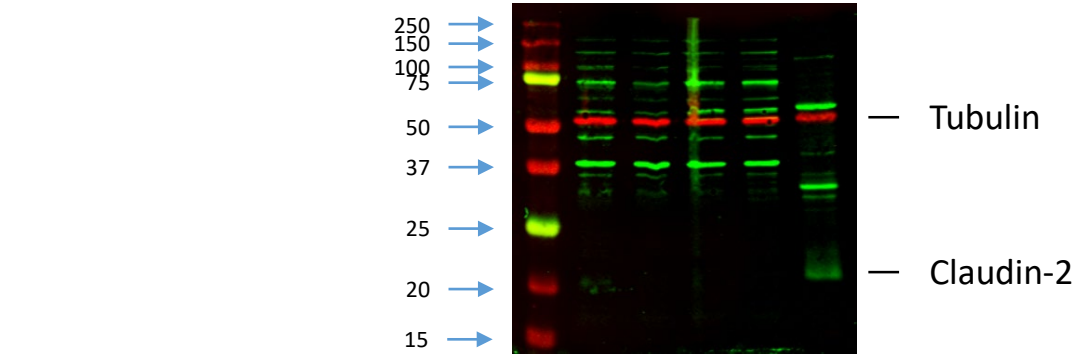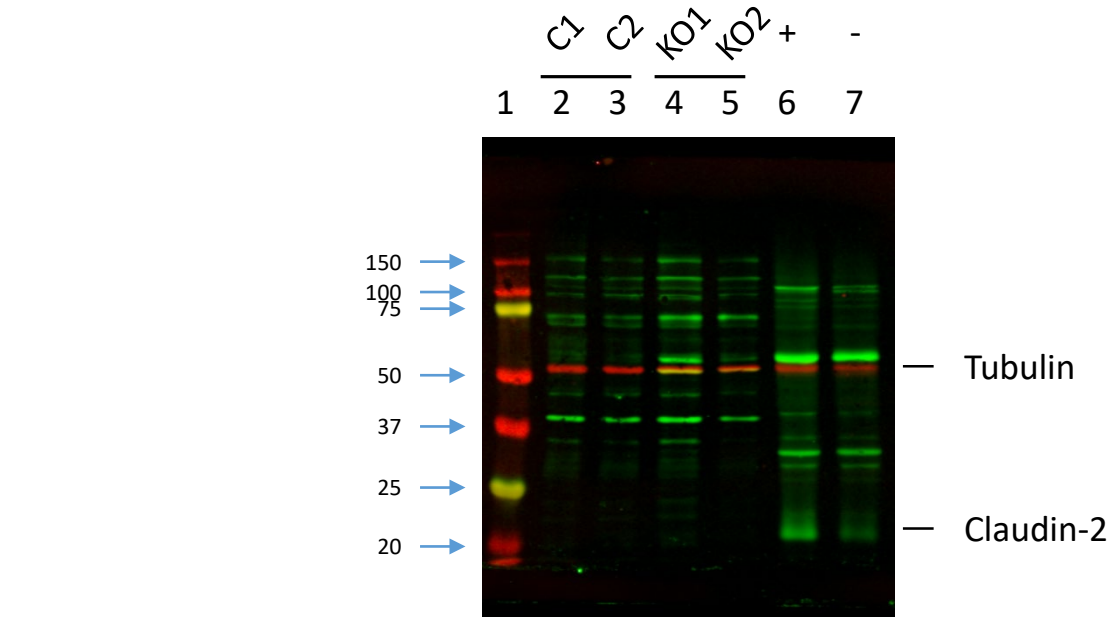

Figure 8B

Claudin-3

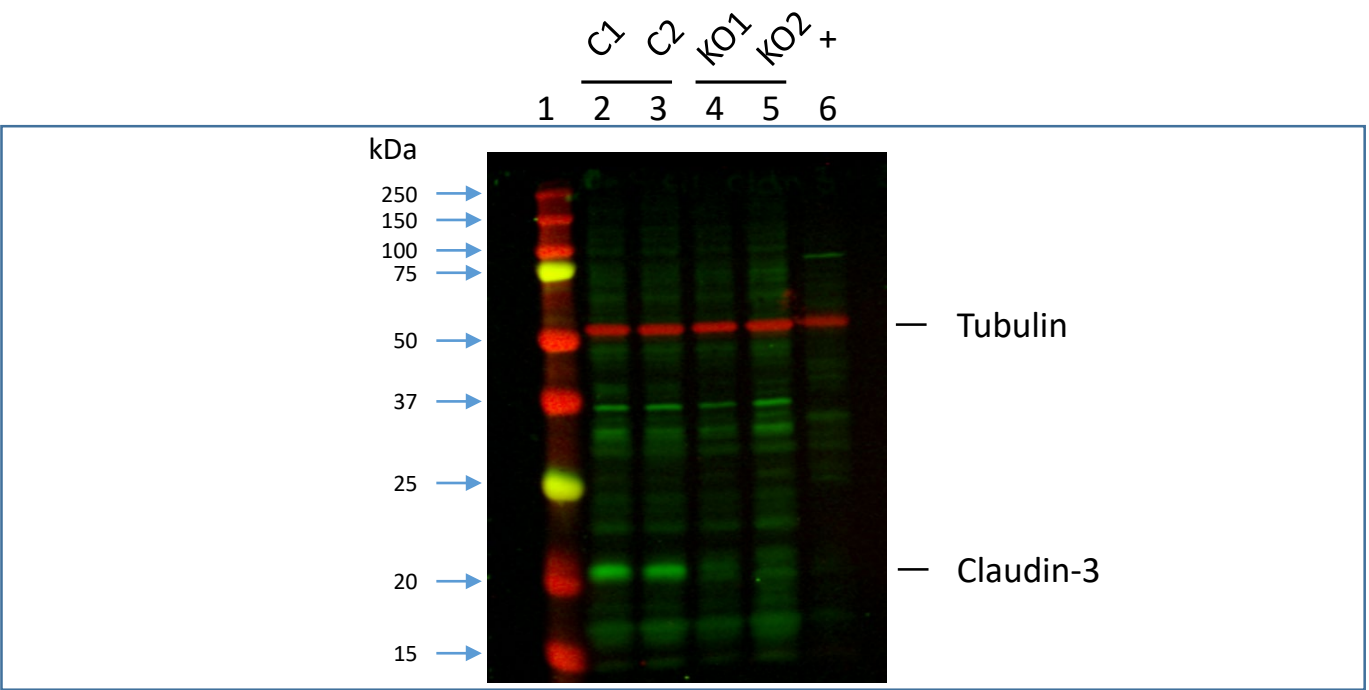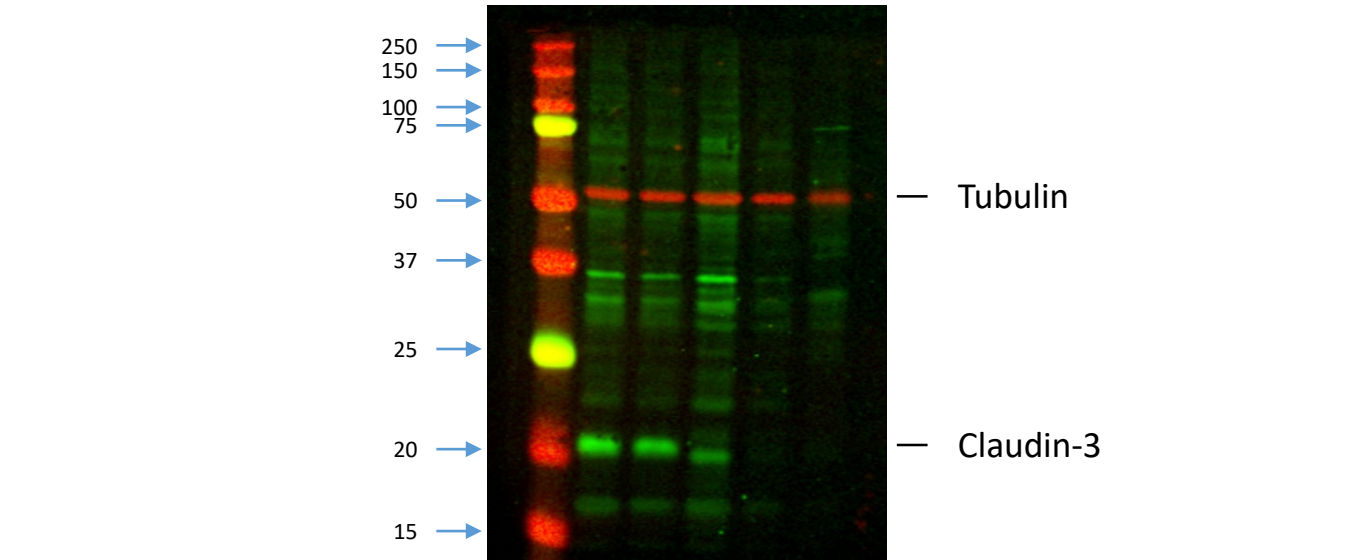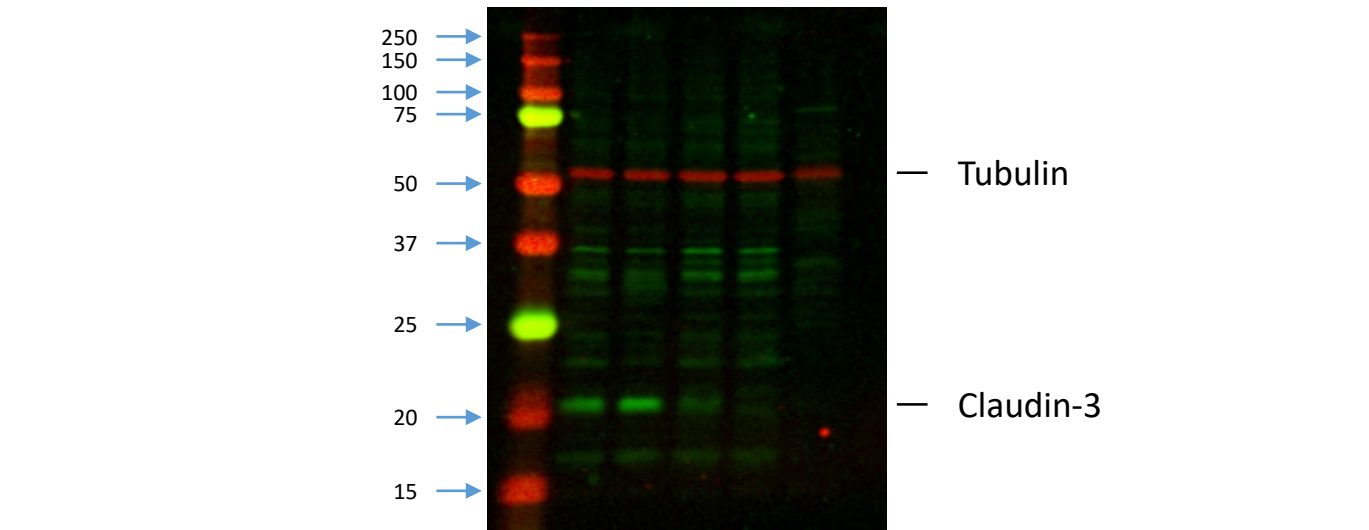

Figure 8B

Claudin-7

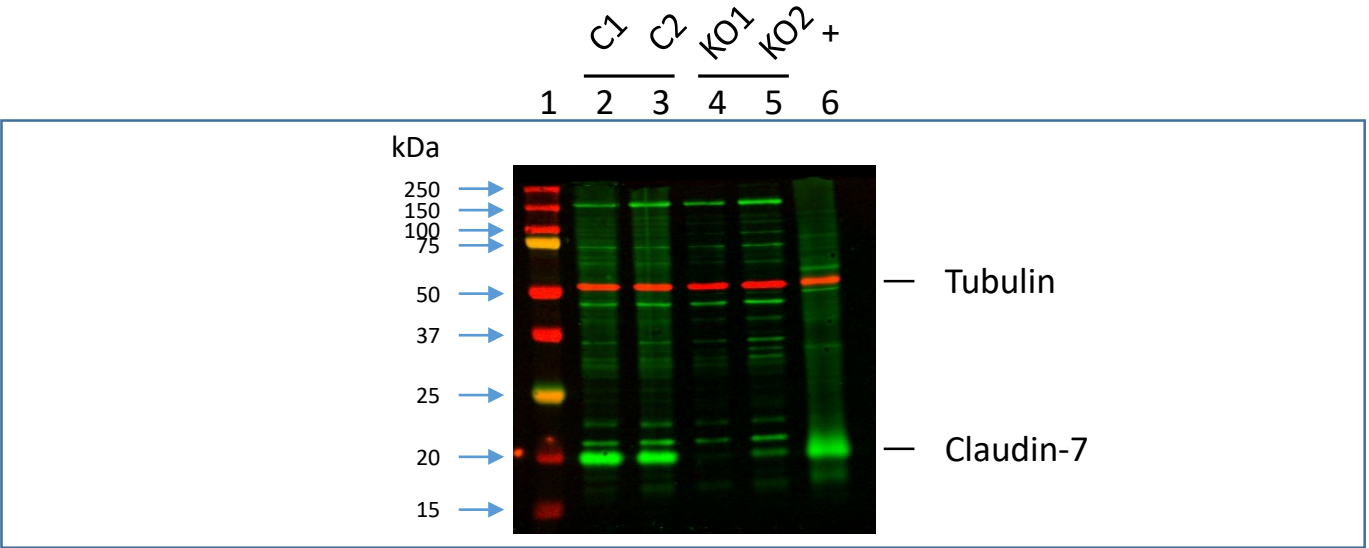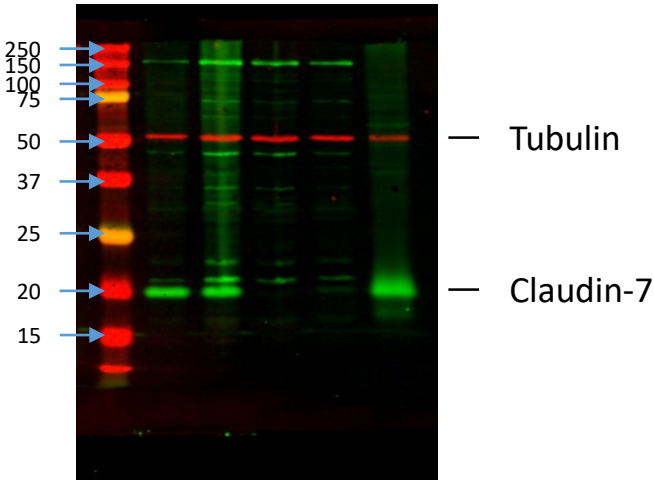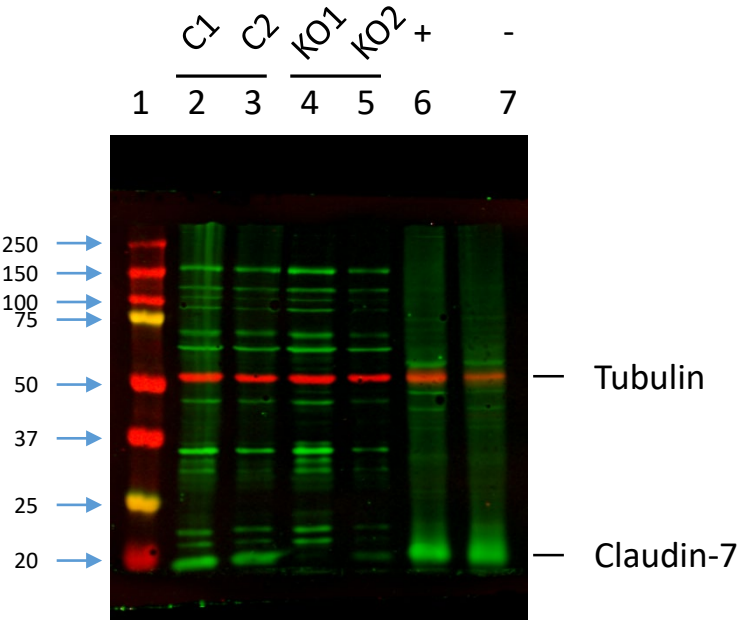

Figure 8B

EpCAM

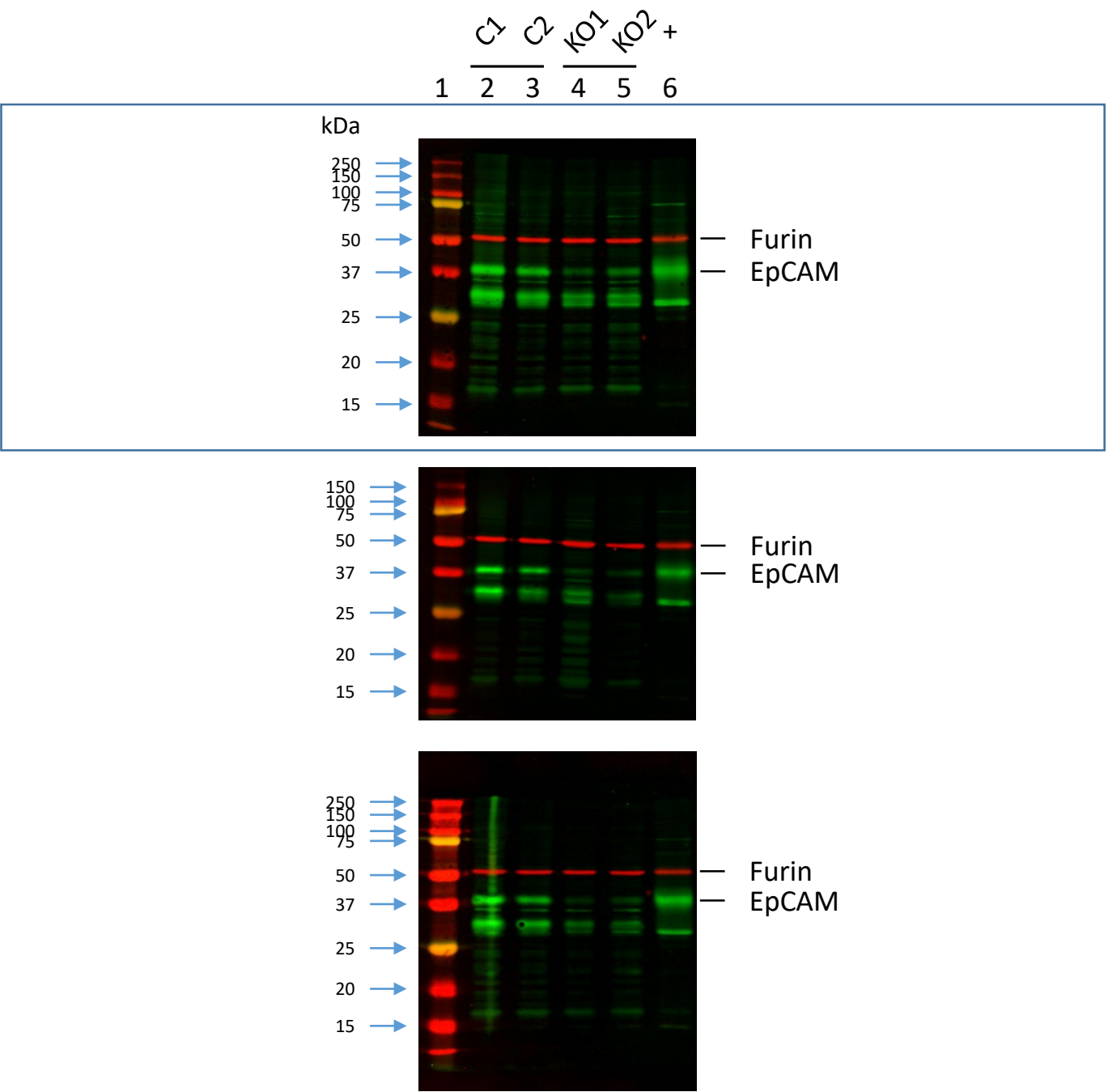

Figure 8E

St14

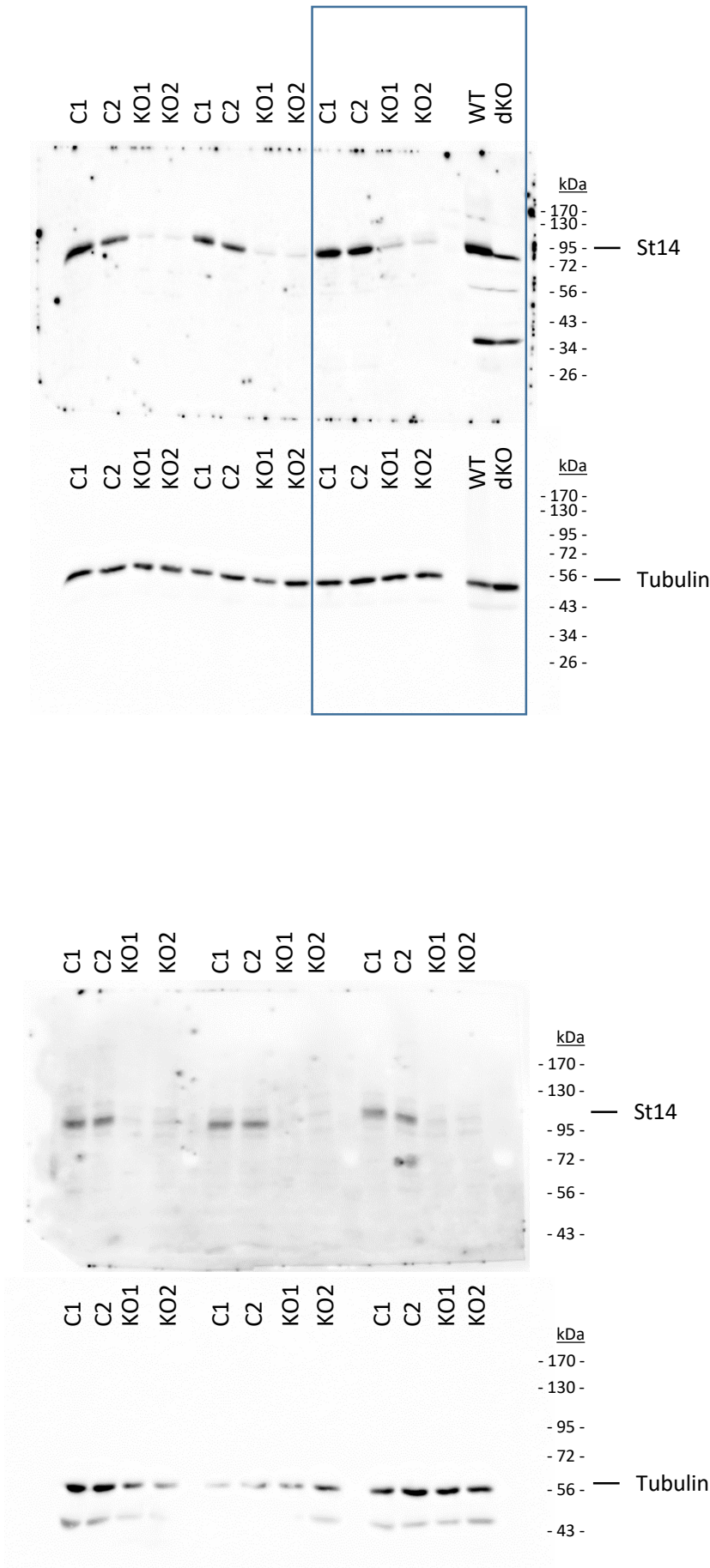

Supplement: Supplementary file 8 [file LSA-2023-02304_SdataF8.1.pdf]

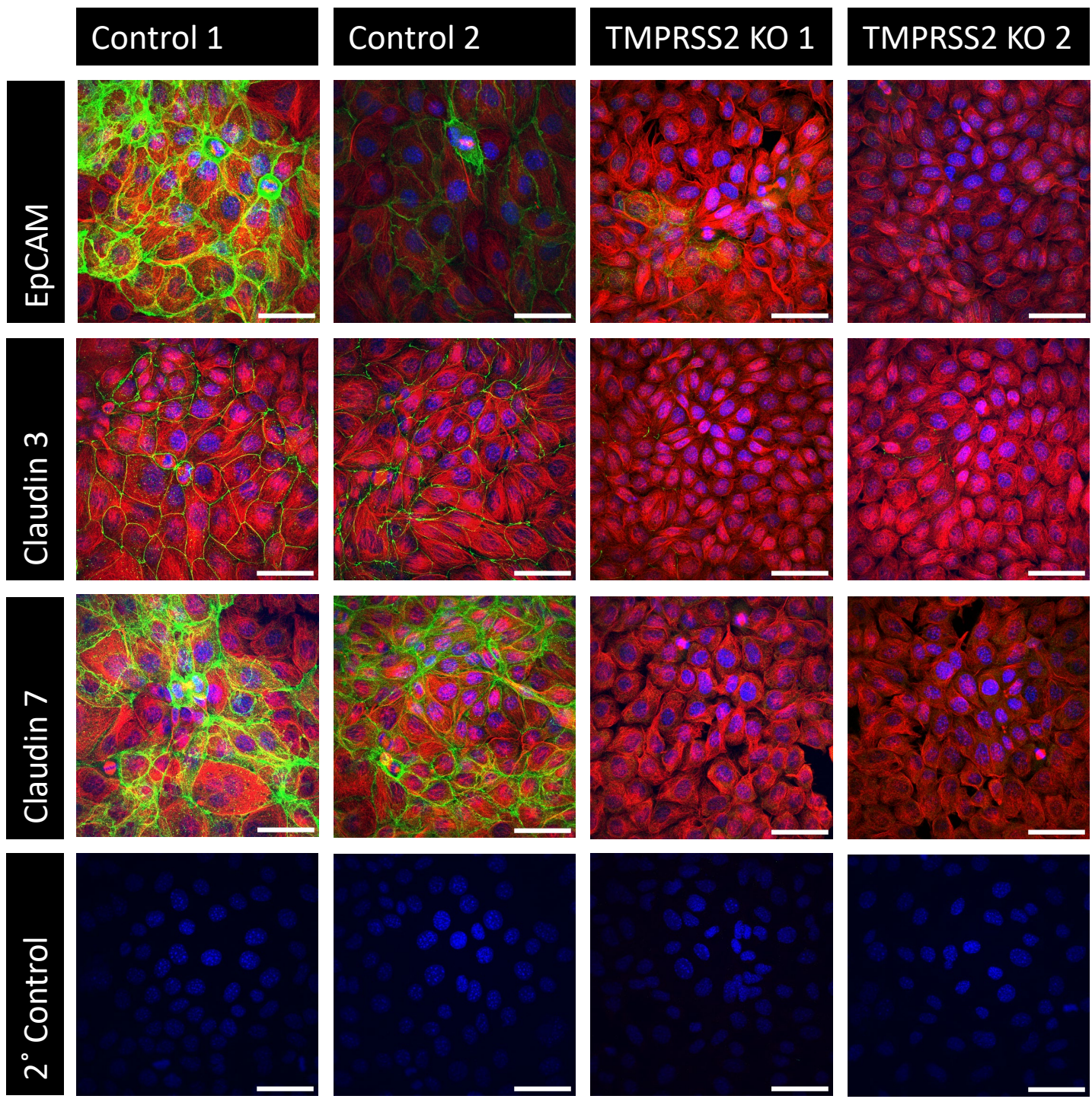

Supplement: Supplementary file 9 [file LSA-2023-02304_SdataF8.2.pdf]
