# Supplementary material for: Tmprss2 maintains epithelial barrier integrity and transepithelial sodium transport
Source: Life Sci Alliance. 2024 Jan 3;7(3):e202302304. doi: 10.26508/lsa.202302304 (PMC10765116; doi:10.26508/lsa.202302304)

Figure 3E

αENaC

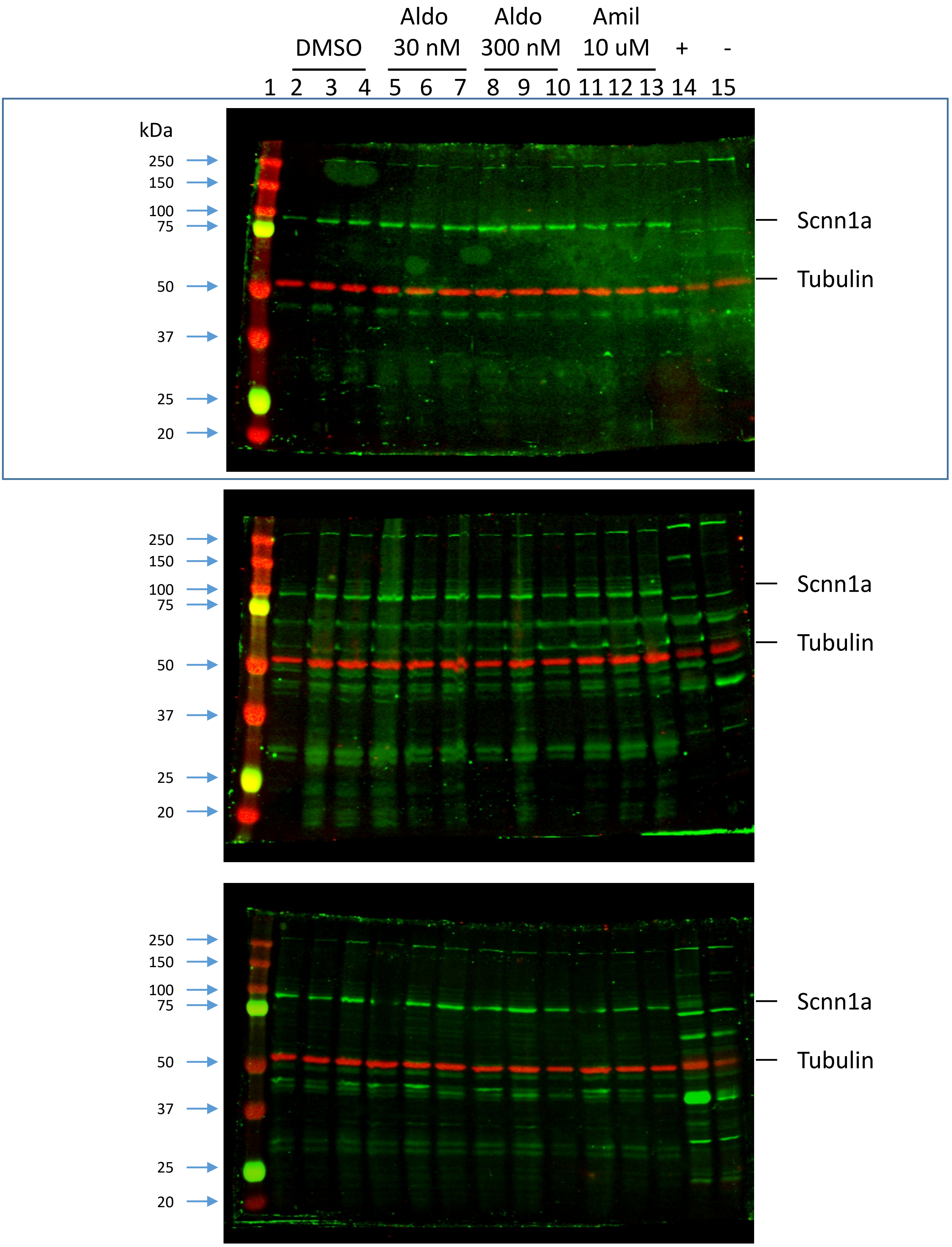

Figure 3E

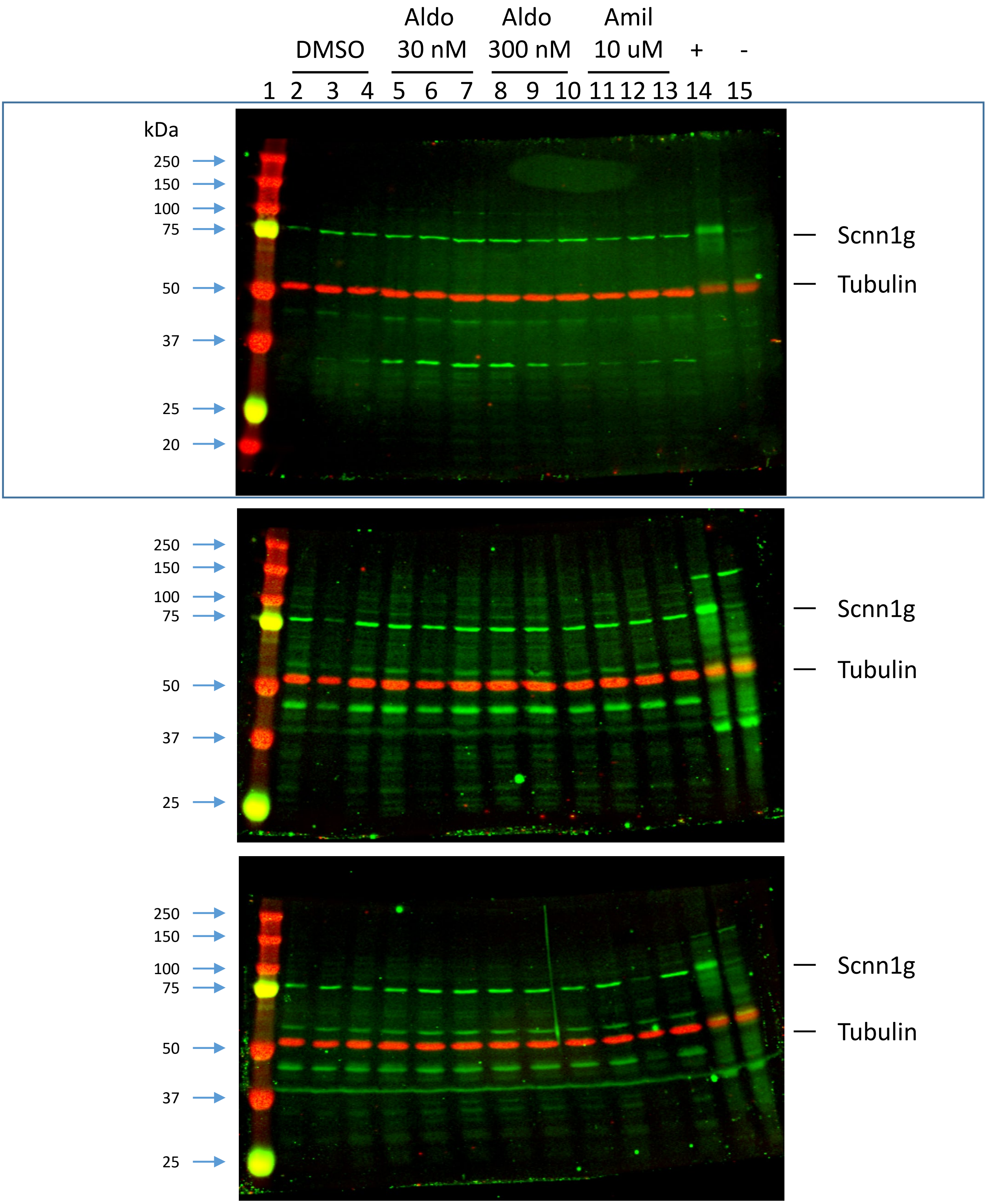
γENaC
Aldo
Aldo
Amil
DMSO
30 nM
300 nM
10 uM
+
-
1 2 3 4 5 6 7 8 9 10 11 12 13 14 15
kDa
250
150
100
75
50
37
25
20
Scnn1g
Tubulin
250
150
100
75
50
37
25
Scnn1g
Tubulin
250
150
100
75
50
37
25
Scnn1g
Tubulin

Figure 3E

furin

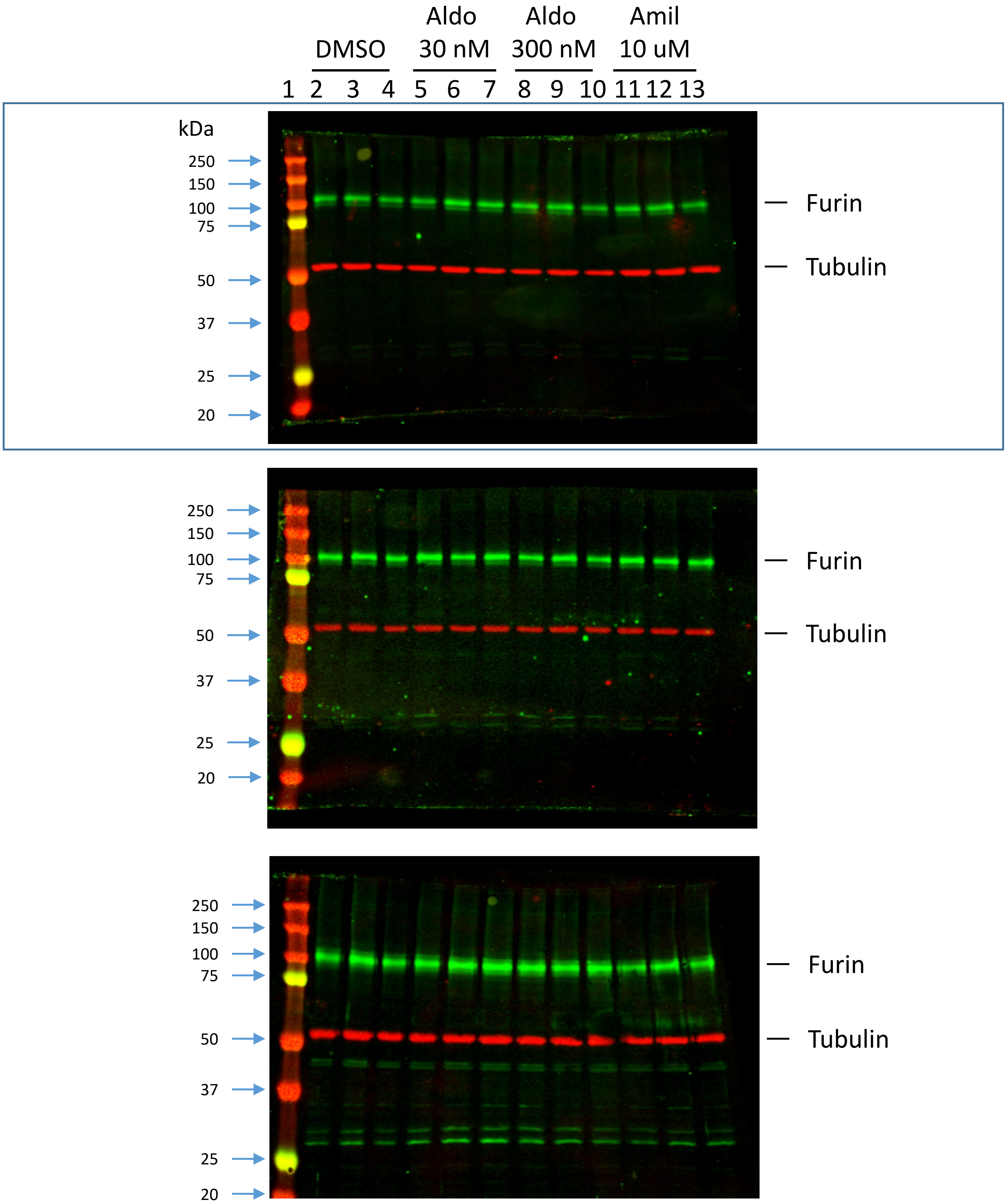

Supplement: Supplementary file 3 [file LSA-2023-02304_SdataF3.pdf]
