# Supplementary material for: Tmprss2 maintains epithelial barrier integrity and transepithelial sodium transport
Source: Life Sci Alliance. 2024 Jan 3;7(3):e202302304. doi: 10.26508/lsa.202302304 (PMC10765116; doi:10.26508/lsa.202302304)
Supplement: Supplementary file 10 [file LSA-2023-02304_SdataFS6.pdf]

Figure S6C

Claudin-3

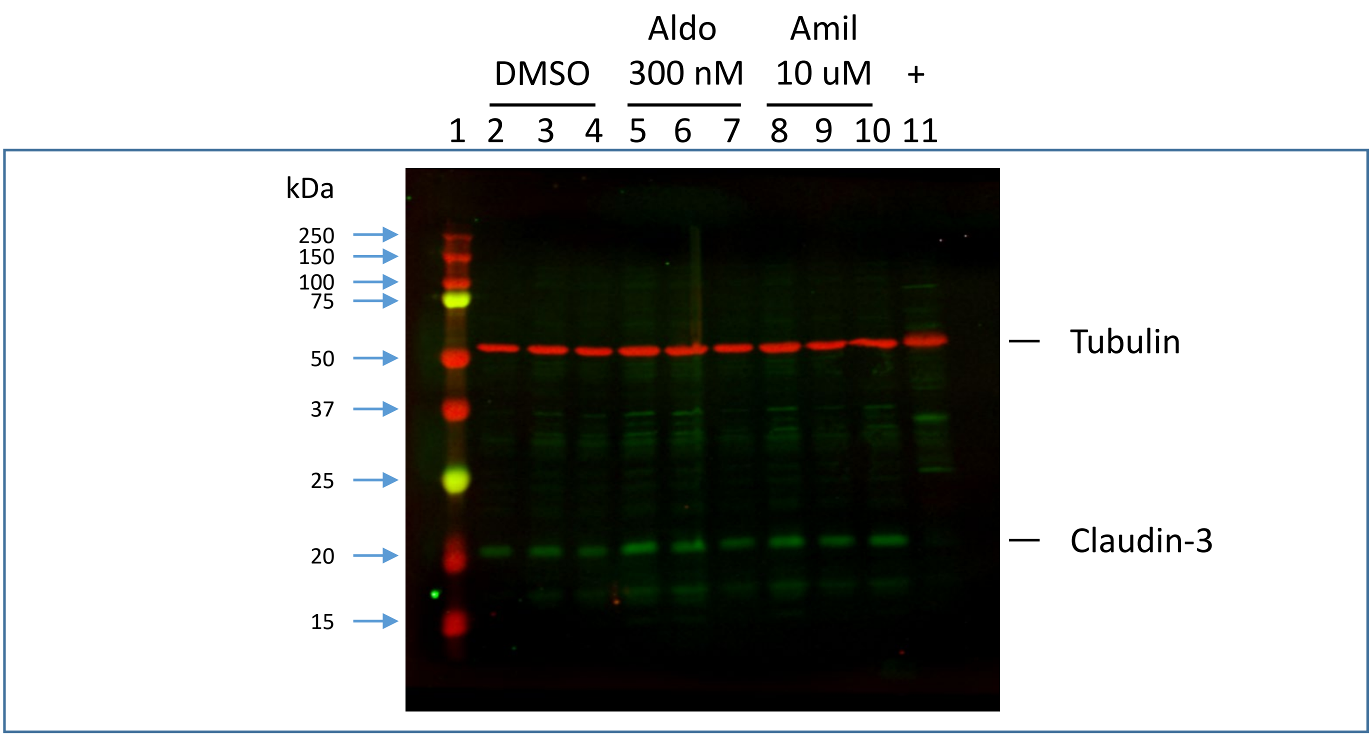

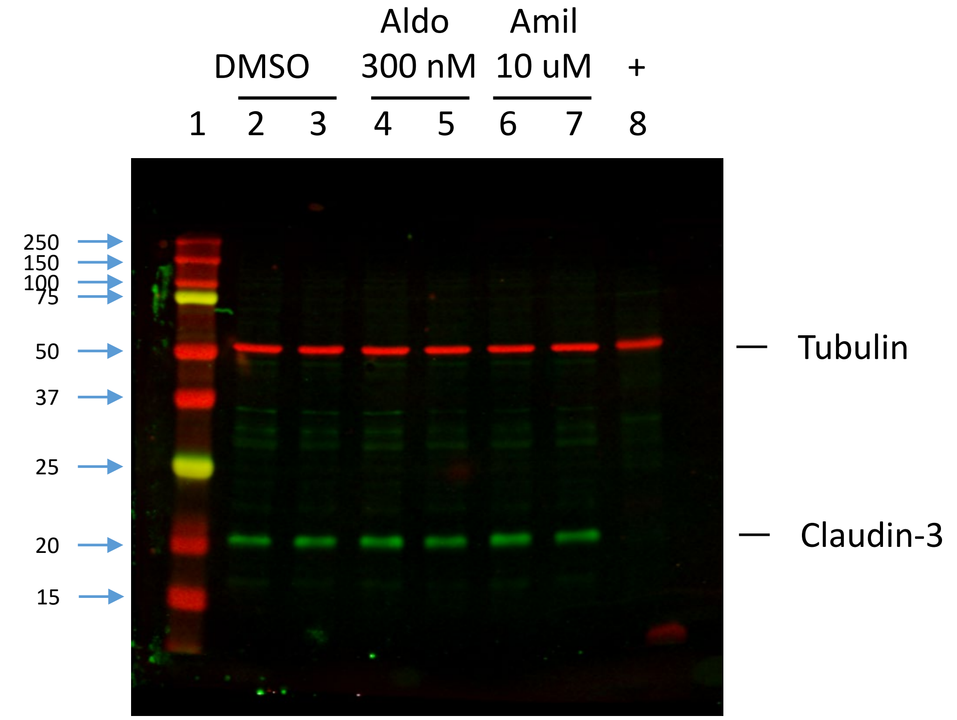

Figure S6C

Claudin-7

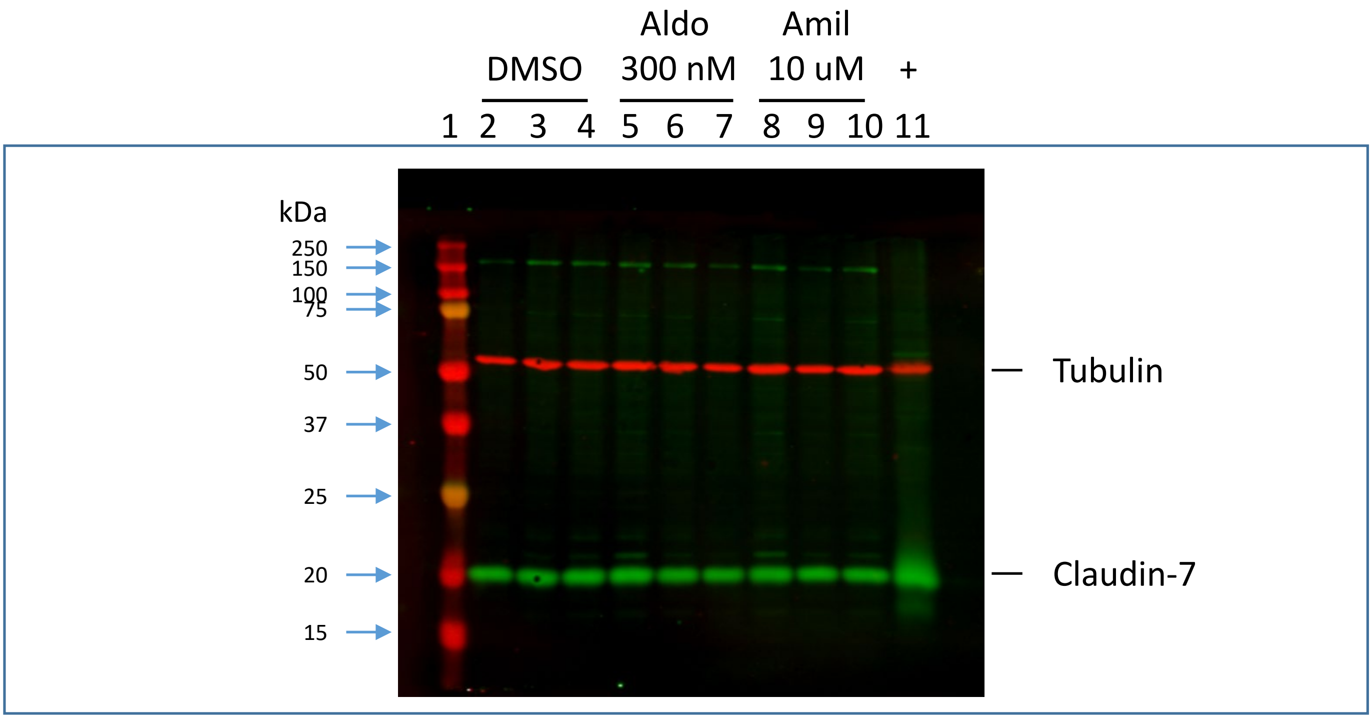

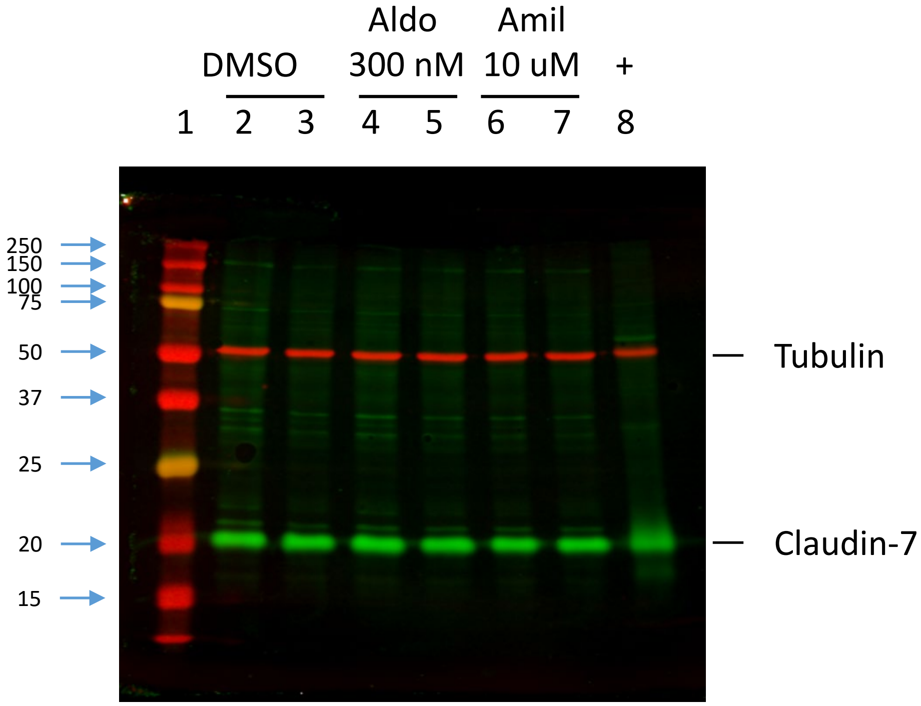

Figure S6C

EpCAM

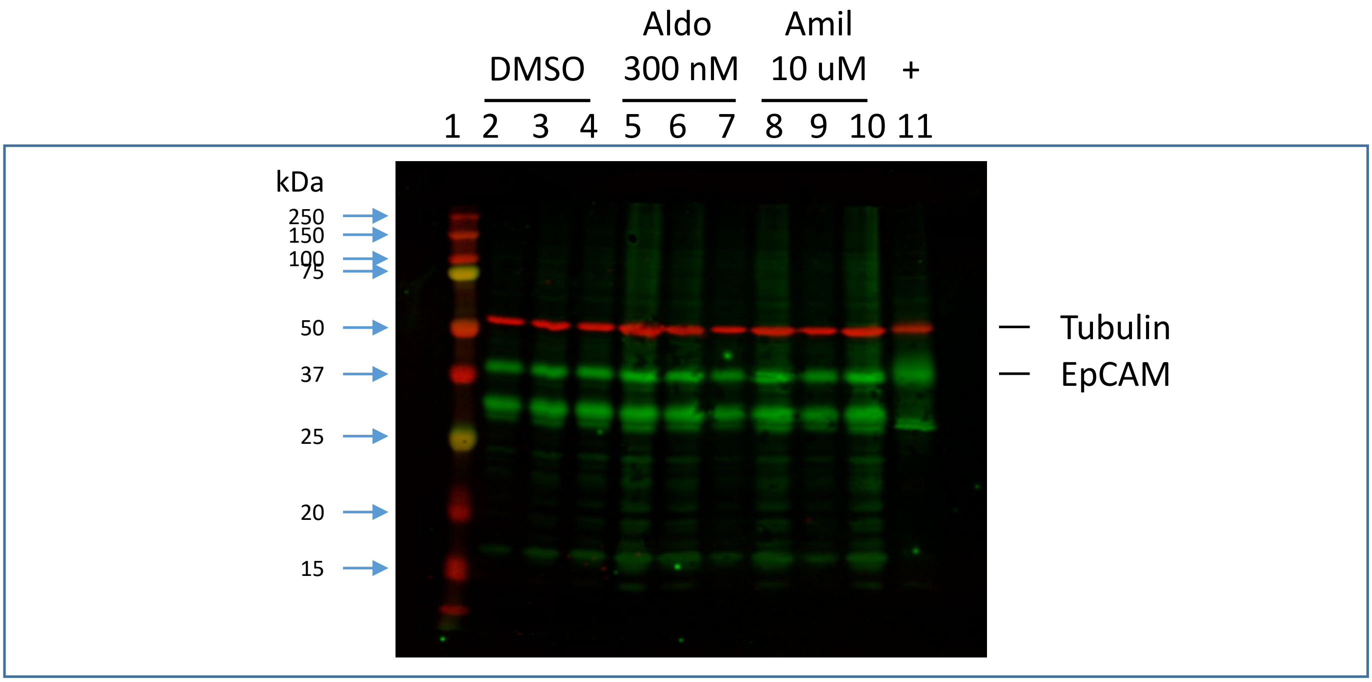

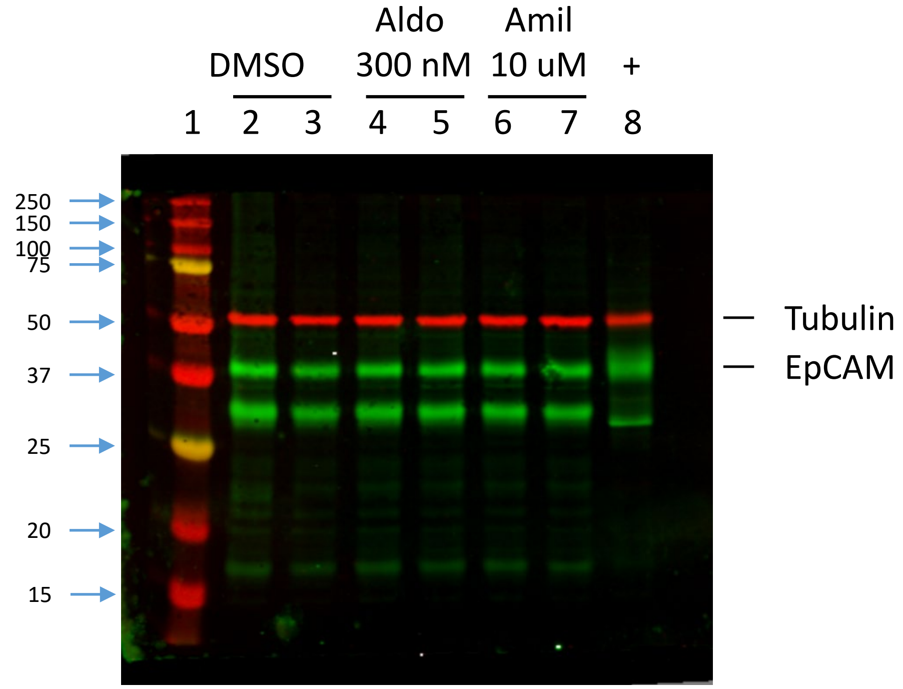

Figure S6F

Claudin-3

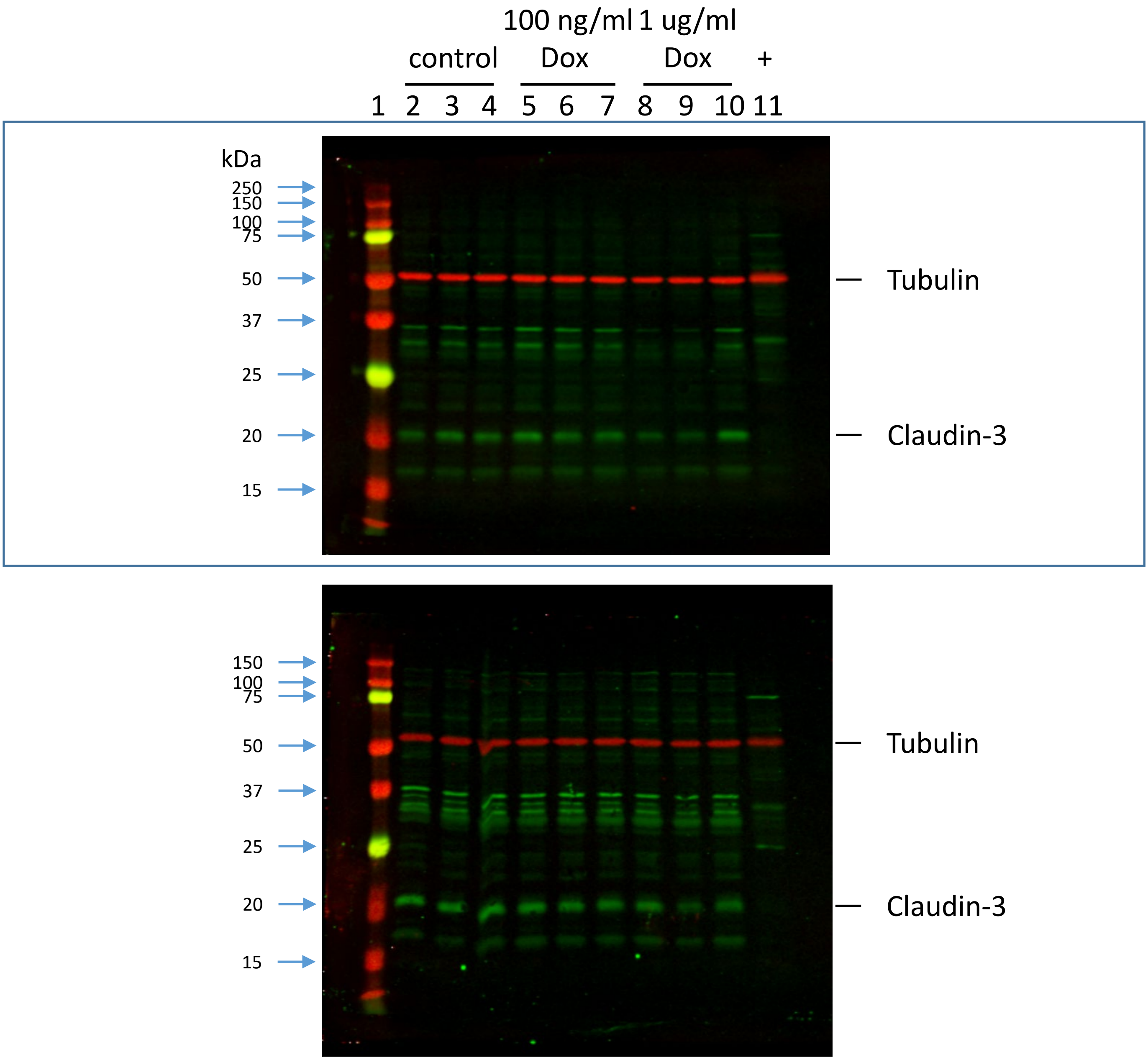

Figure S6F

Claudin-7

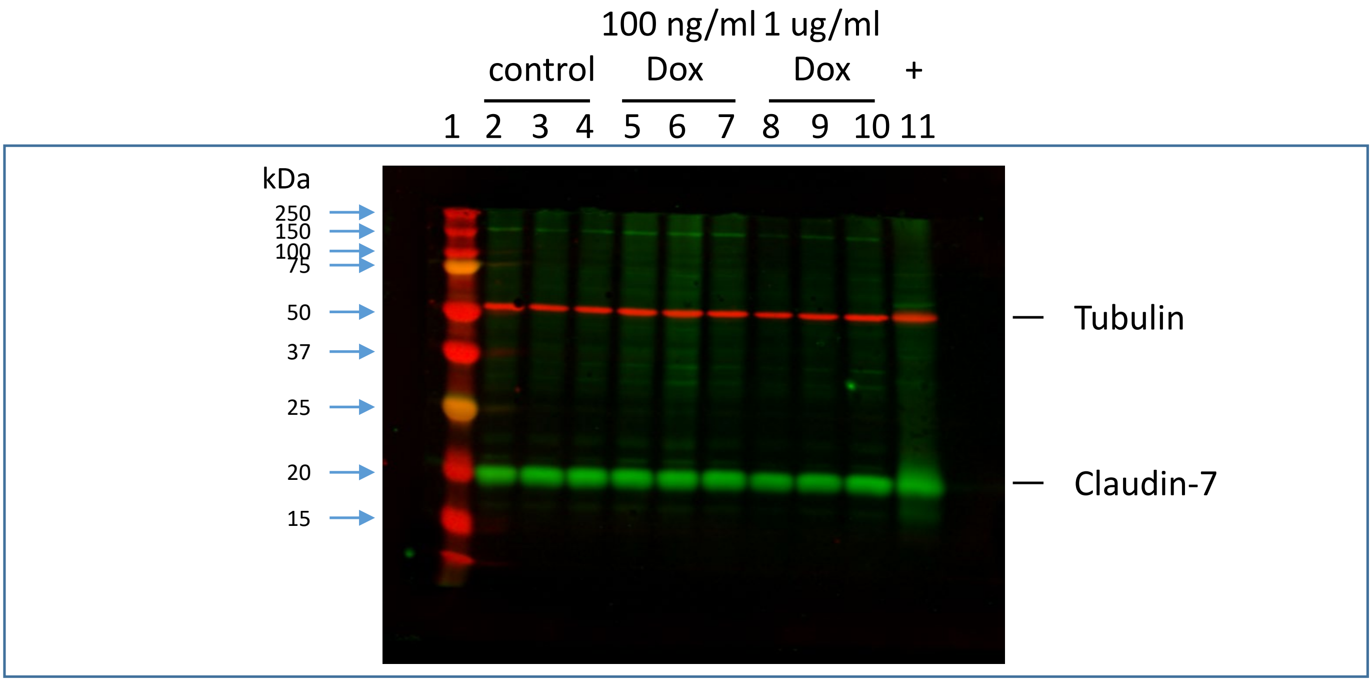

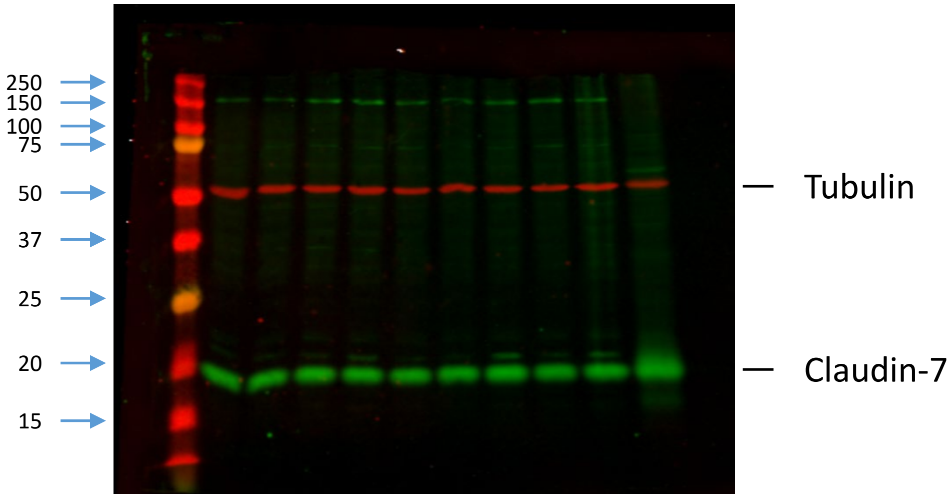

Figure S6F

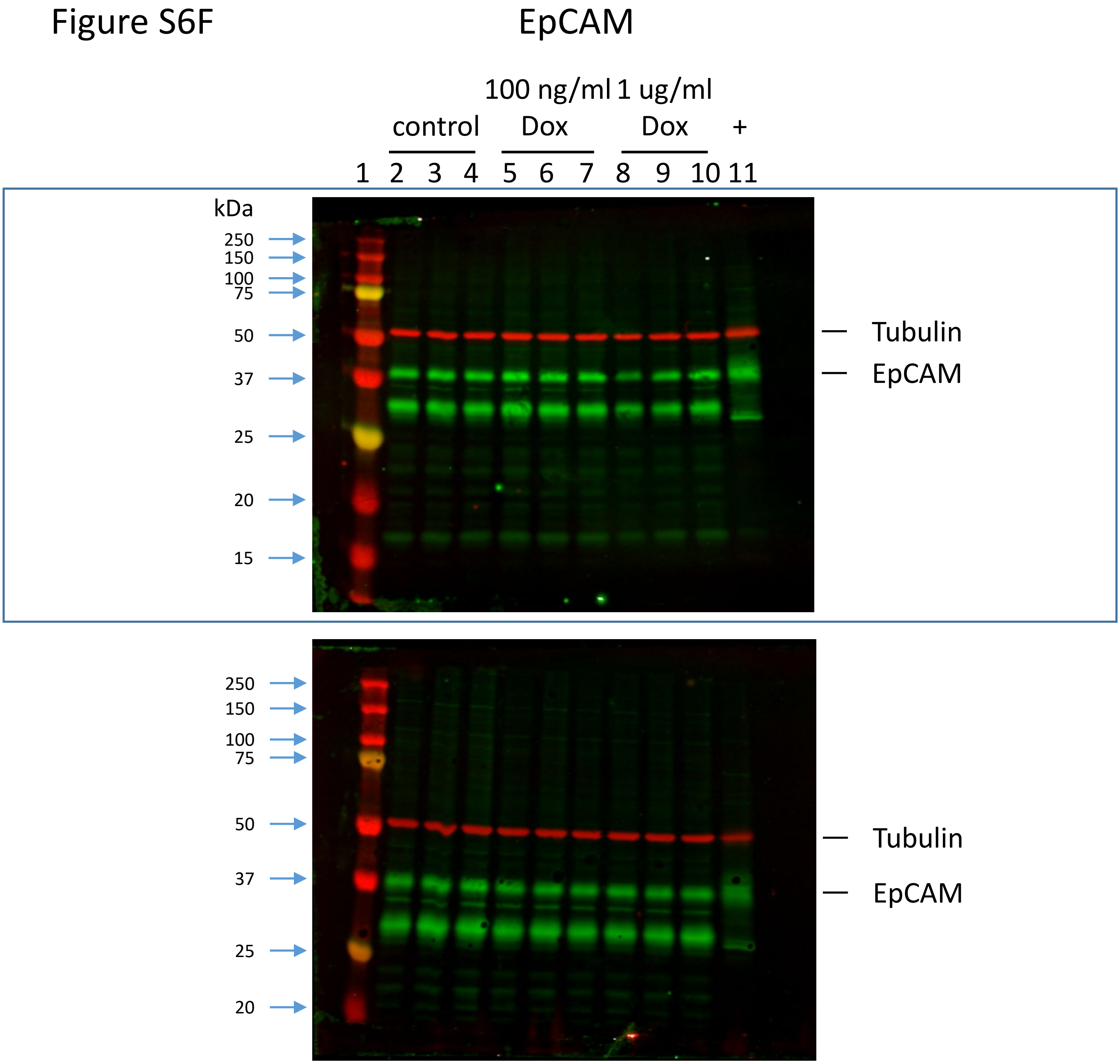
EpCAM
100 ng/ml
1 ug/ml
control
Dox
Dox
+
1 2 3 4 5 6 7 8 9 10 11
kDa
250
150
100
75
50
37
25
20
15
Tubulin
EpCAM
250
150
100
75
50
37
25
20
Tubulin
EpCAM
